# Relationship between nanoscale assembly and cytotoxicity in the piperazine amides of betulinic acid: an *in vitro* study

**DOI:** 10.1039/d6md00638h

**Published:** 2026-09-08

**Authors:** Martina Wimmerová, Miroslav Šlouf, Lucie Rárová, Marie Kvasnicová, David Šaman, Zdeněk Wimmer

**Affiliations:** a Department of Chemistry of Natural Compounds, University of Chemistry and Technology in Prague CZ-16628 Prague Czech Republic zdenek.wimmer@vscht.cz; b Institute of Macromolecular Chemistry, Czech Academy of Sciences CZ-16206 Prague Czech Republic; c Laboratory of Growth Regulators, Faculty of Science, Palacký University & Institute of Experimental Botany, Czech Academy of Sciences CZ-77900 Olomouc Czech Republic; d Institute of Organic Chemistry and Biochemistry, Czech Academy of Sciences CZ-16610 Prague Czech Republic

## Abstract

Six new piperazine amides of betulinic acid, partly decorated with a glycine unit, were synthesized and purified and their structures were elucidated. This study explored whether relationships could be identified among the molecular structures, nanoscale self-assembly, and cytotoxicity of the synthesized compounds. Because of the limited quantities of the target compounds finally available for the analytical methods, their selection had to be very careful. NMR spectroscopy and mass spectrometry were used to elucidate the molecular structures, while transmission electron microscopy (TEM) was employed to characterize the supramolecular structures formed after dissolution in methanol and water. The target compounds reproducibly displayed several basic morphologies depending on the solvent type (methanol *vs.* water) and the time in solution (1 h *vs.* 48 h). Several characteristic morphologies were observed, including cloud-like assemblies, isometric nanoparticles of various sizes, fibrous nanostructures with thicknesses below 10 nm, and combinations of these structures, depending on the compound, solvent, and time in solution. The target compounds (6 and 8), the parent compound (1) and the intermediates (2 and 4) were subjected to cytotoxicity assays in the HeLa and MCF7 cancer cell lines while using normal human fibroblasts (BJ) as the reference cells. Cisplatin (*cis*-diaminedichloroplatinum(ii), **CDDP**), a pharmacologically used anticancer agent, was used for comparing the cytotoxicity levels and cytotoxicity profiles of the studied compounds. Compounds 6 and 8 were specifically examined for possible associations between nanoscale self-assembly and cytotoxicity studied *in vitro*. The results provided initial insights into the complex relationship among the molecular structures, self-assembly, and cytotoxicity of the studied compounds.

## Abstract 2

Structures, cytotoxicity data and TEM micrographs of the new target compounds are presented, showing the differences in the cytotoxicity and nanoscale self-assembly of the studied compounds.

## Graphical abstract

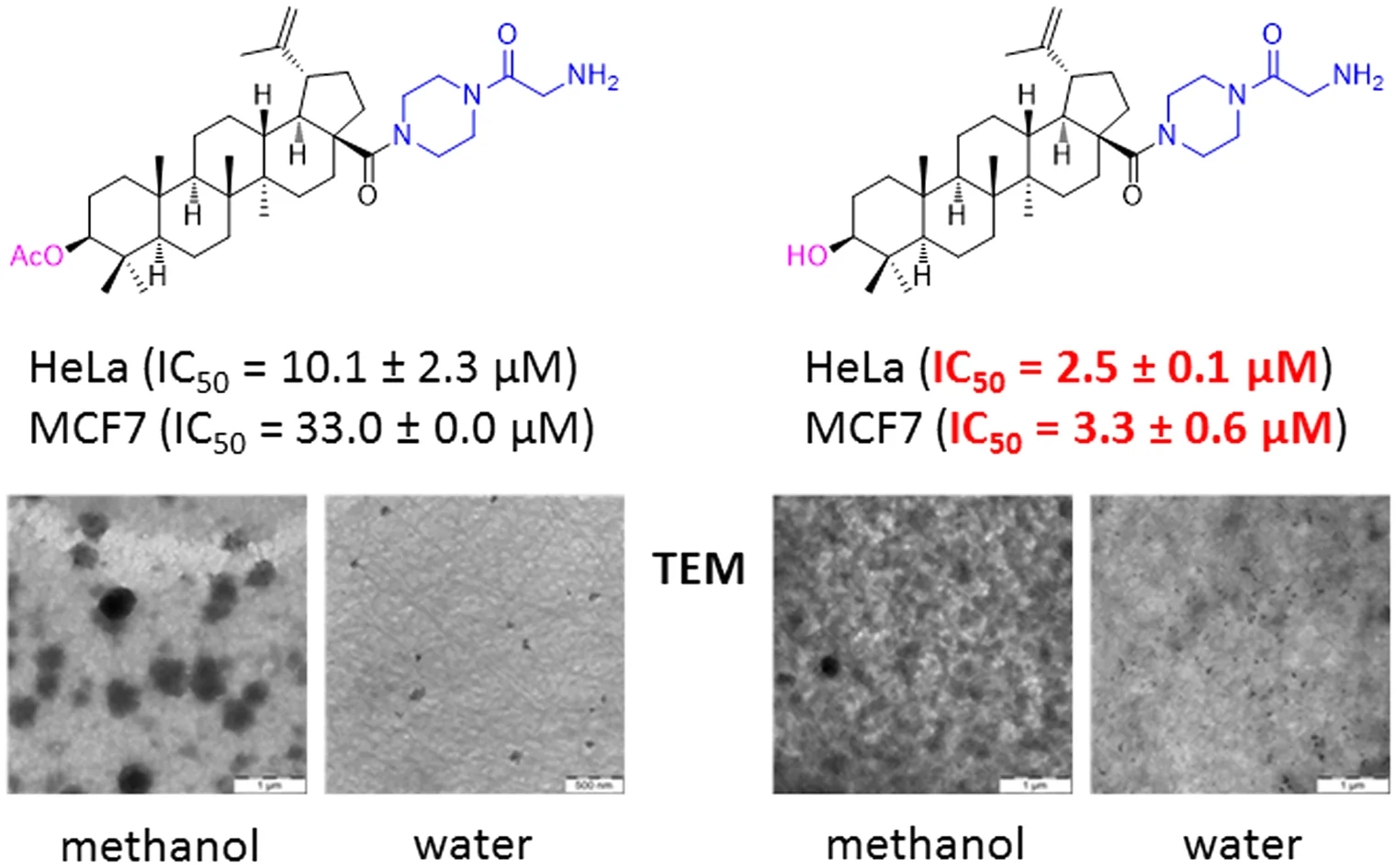

## 1. Introduction

Small molecules based on natural plant products have often been subjected to nanoscale assembly. The nanostructured materials display various physicochemical characteristics. They are applicable in various fields of investigation, namely, in pharmacology, medicine, and different technical areas.^1^ The capability of a nanoscale assembling of triterpenoids, secondary plant metabolites, selected among many plant products, has been studied in different organic solvents and in aqueous media.^2–4^ The semisynthetic derivatives of triterpenoids have also been included in this investigation.^2–4^ Subsequently, the investigation of the ability to display biological activity usually represented an accompanying objective in the complex studies of the above plant products and of their semisynthetic derivatives.^2–4^ During the current investigation program, numerous natural triterpenoids and their different semisynthetic derivatives were studied in searching for the convenient compounds capable of displaying required characteristics.^2^ Based on the experience resulting from a recent investigation, the derivatives of ursolic acid seem to be the most potent ones so far in terms of their capability for nanoscale assembly among the compounds derived from other frequently studied pentacyclic triterpenoids.^4^ In contrast, only a limited number of the studied derivatives of betulinic acid has displayed these characteristics thus far.^2^ However, different biological activities, such as cytotoxicity, antiviral activity and/or antimicrobial activity, have been displayed by betulinic acid derivatives quite often.^5–8^ We have already documented a relationship between the cytotoxicity and the dynamic nanoscale assembly of the studied compounds.^9^ Generally, nanoscale materials have already been proven to affect the biological activity of drugs either positively or negatively, and they may positively assist in the targeted transport of biologically active drugs to the active sites of receptors to augment and accelerate the mode of action of the drugs.^10,11^

Betulinic acid [(3β)-3-hydroxylup-20(29)-en-28-oic acid; 1; Scheme 1] is the pentacyclic triterpenoid of the lupane family, and it belongs among the secondary plant metabolites.

**Scheme 1:**
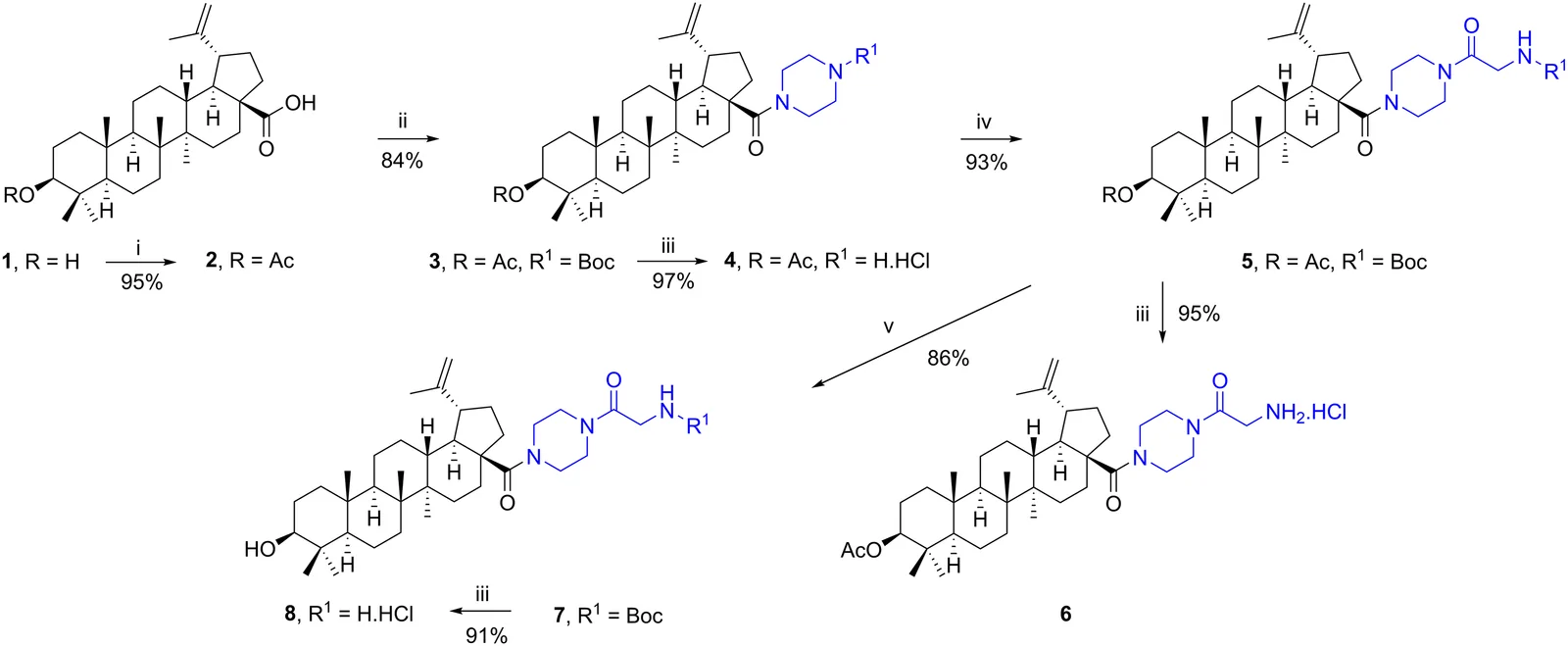
Synthetic procedure: (i) acetic anhydride, DIPEA, DMAP, and THF; (ii) (a) oxalyl chloride and DCM; (b) *N*-Boc-protected piperazine, DIPEA, and DCM in an argon atmosphere; (iii) HCl (gas) in 1,4-dioxane; (iv) *N*-Boc-glycine, HOBt, NMM, EDC·HCl, and DCM in an argon atmosphere; and (v) LiOH·H_2_O, and methanol.

Betulinic acid and its wide spectrum of semisynthetic derivatives have been reviewed several times in recent years.^12–15^ These investigations have documented the spectrum of biological effects of betulinic acid and its derivatives, but there are still relatively rare reports on their physicochemical characteristics, including nanoscale assembly.^16^ Piperazine belongs to the class of polyamines. This cyclic diamine is often considered a biologically active molecule, such as in functional antibiotics or serotonin antagonists acting at the serotonin receptors^17,18^ or in the recently designed cytotoxic drugs and compounds showing the topoisomerase I inhibition effect.^19–21^ Glycine is the simplest, achiral and stable proteinogenic amino acid. It displays different biological effects, including the effect of the neurotransmitter inhibitor in the central nervous system or the effect in the postsynaptic potential.^22^ It often forms conjugates with both natural compounds, for increasing their solubility, and xenobiotics, for their detoxification.^23^

The investigation reported herein involved betulinic acid, piperazine and glycine to search for novel structures capable of nanoscale assembly and displaying cytotoxicity. A potential application of the prepared nanostructured materials consists of a combination of their ability to form nanoscale assemblies and their ability to display biological activity. Therefore, the objectives of this investigation consisted of several partial tasks: (a) synthesis and structure elucidation of the piperazine conjugates with betulinic acid, partly decorated by the glycine unit, the incorporation of which brings an additional amide bond to the target molecules; (b) the investigation of the nanoscale assembly of the target compounds 6 and 8 by transmission electron microscopy (TEM) performed in two independent solvents at the time of 1 h and 48 h from the point of complete dissolution of the target compounds 6 and 8; (c) cytotoxicity screening tests involving the target compounds and the intermediate products obtained during the synthetic procedure to confirm potential advantages of the complex target structures on cytotoxicity; and (d) the evaluation of the relationship between the nanoscale assembly of the target compounds 6 and 8 on their observed cytotoxicity.

## 2. Results and discussion

### 2.1. Synthetic procedure

The synthesis of the target compounds started with the protection of the C(3)–OH group of 1 by converting it into the C(3)-acetate derivative 2 using acetic anhydride in THF in the presence of a base (DIPEA) and a reaction accelerator (DMAP; path i; Scheme 1). The new amide bond in the C(17)–COOH group was then formed in a two-step reaction. The carboxyl group was converted into its acyl chloride form using oxalyl chloride in DCM. The acyl chloride was then reacted with the *N*-Boc-protected piperazine derivative in DCM using DIPEA as a base, yielding 3 (path ii). The Boc-protecting group in 3 was removed using a 4 M solution of HCl (gas) in 1,4-dioxane, affording 4 (path iii). The free amino group of 4 was reacted with the *N*-Boc-protected glycine using 1-hydroxybenzotriazole (HOBt) and *N*-methylmorpholine (NMM) in dichloromethane (DCM), followed by the addition of *N*-ethyl-*N′*-(3-dimethylaminopropyl)carbodiimide hydrochloride (EDC·HCl) under an argon atmosphere, affording product 5 (path iv). The next step of this synthesis consisted of removing the Boc-protecting group present in 5 using the procedure described above (path iii), yielding 6, one of the target products. The other target product 8 was also obtained from the intermediate product 5. It was first treated with lithium hydroxide in methanol to liberate the C(3)–OH group, yielding 7 (path v). The Boc-protecting group in 7 was removed using a 4 M solution of HCl (gas) in 1,4-dioxane (path iii), yielding 8. Most of the synthetic methods described above have already been used in a combination with several other plant triterpenoids during the previous investigation performed by this research team.^4,24^

### 2.2. Nanoscale assembly

The ability of both the target compounds (6 and 8) to form nanoscale assemblies was monitored by transmission electron microscopy (TEM). Compounds 6 and 8 were soluble in both methanol and water, and the TEM studies were performed in both these solvents (Fig. 1). Methanol was selected due to its miscibility with organic solvents and water, and water was selected due to its compatibility with the physiological environment and suitability for potential pharmacological and/or medicinal applications. During the TEM sample preparation, the dissolved compounds were deposited on the electron-transparent carbon film, and the excess solvent was removed instantly using the fast-solvent removal protocol, which resulted in the reproducible fixing of the nanoscale assemblies on the film, as documented in several previous studies.^25–27^

**Fig. 1:**
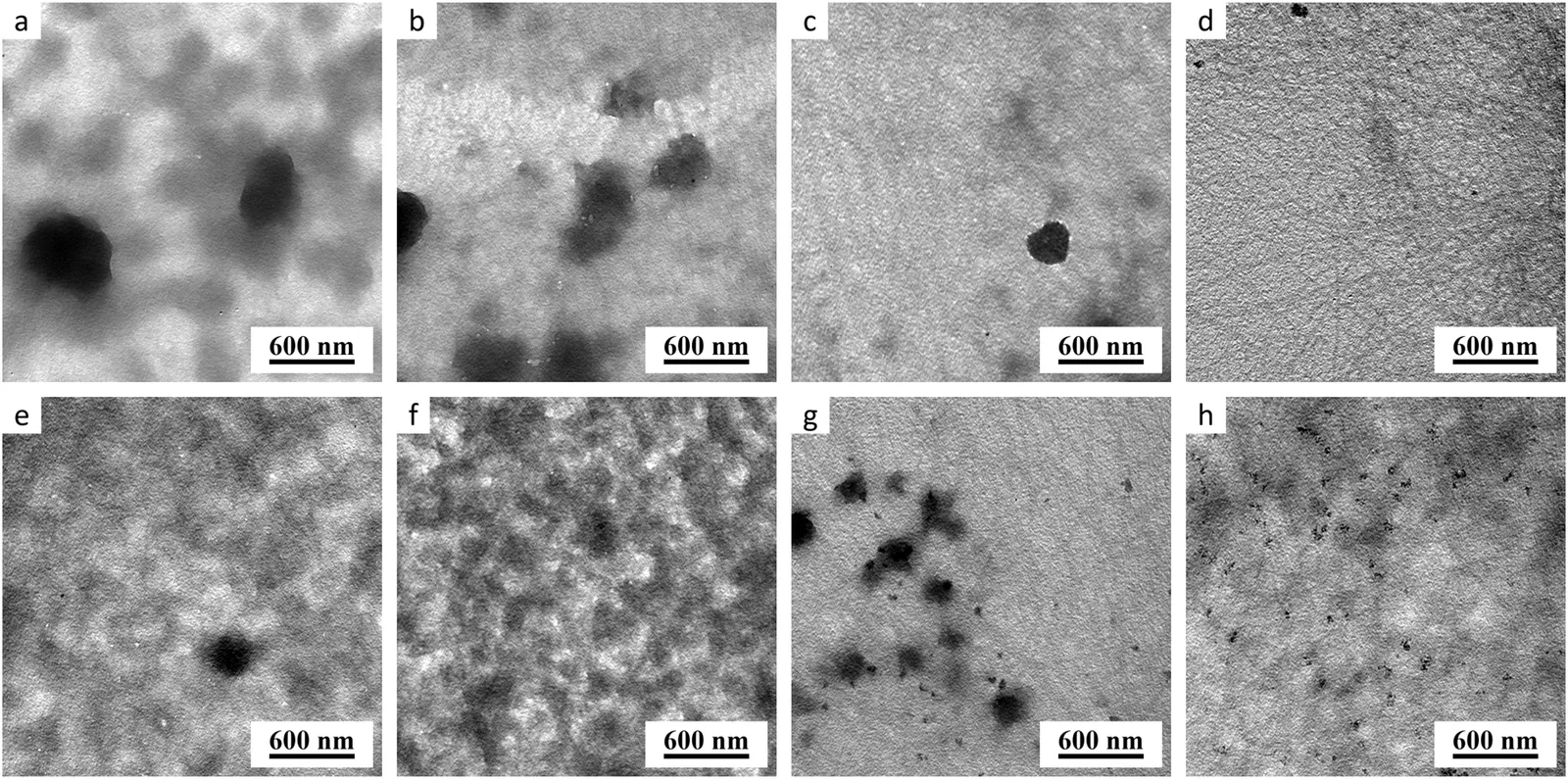
TEM micrographs showing the formation of nanoscale assemblies of 6 and 8 in methanol and water. (a) 6, methanol, 1 h; (b) 6, methanol, 48 h; (c) 6, water, 1 h; (d) 6, water, 48 h; (e) 8, methanol, 1 h; (f) 8, methanol, 48 h; (g) 8, water, 1 h; and (h) 8, water, 48 h. The time of imaging (1 h and 48 h) was measured from the point of complete dissolution of compounds 6 and 8.

Compound 6 formed free, diffuse, cloud-like nanoscale assemblies combined with isometric nanoparticles (200–400 nm) that were found in methanol at the time of 1 h (Fig. 1a). They were slowly transformed into a mixture of isometric nanoparticles and long fibrous nanostructures with thicknesses below 10 nm at the time of 48 h (Fig. 1b). In water, compound 6 formed a mixture of free cloud-like nanoscale assemblies and long fibrous nanostructures at the time of 1 h (Fig. 1c). The appearance of isometric nanoparticles was rare. At the time of 48 h in water, compound 6 existed as a nice fibrous nanomaterial (Fig. 1d). The behaviour of compound 8 was different. In methanol, it showed a mixture of cloud-like nanoscale assemblies and long fibrous nanostructures at the time of 1 h (Fig. 1e), which was transformed into more pronounced cloud-like nanoscale assemblies connected by less clearly visible fibrous nanostructures at the time of 48 h (Fig. 1f). In water, compound 8 showed an analogous mixture of big isometric nanoparticles (∼200 nm) and fibrous nanomaterials at the time of 1 h (Fig. 1g). The morphology later changed in favour of more isometric nanoparticles but with much smaller sizes (20–40 nm) connected by less intensively pronounced long fibrous nanostructures at 48 h (Fig. 1h). For additional details, refer to the SI (section 1.2. and Fig. S1 and S2).

### 2.3. Cytotoxicity screening tests

The cytotoxicity screening tests involved the target compounds 6 and 8, the intermediate products 2 and 4 and the parent compound betulinic acid (1). The spectrum of the compounds subjected to cytotoxicity assays also included the pharmacologically used anticancer agent cisplatin (*cis*-diaminedichloroplatinum(ii), **CDDP**; Table 1). The results of cytotoxicity assays performed in the HeLa (cervical cancer) and MCF7 (breast adenocarcinoma) cancer cell lines showed that the cytotoxicity of compound 8 surpassed that of compound 6 in both cancer cell lines (Table 1). In our previous studies that were focused on different amide derivatives of several triterpenoids, mostly less polar compounds displayed higher cytotoxicity than the more polar ones, as documented recently.^4^ Compound 6 showed a better cytotoxicity profile than cisplatin (**CDDP**). Betulinic acid (1) was inactive within the tested range of concentrations of the studied compounds. In turn, both intermediate compounds (2 and 4) showed cytotoxicity. Although 2 displayed mild cytotoxicity in both cancer cell lines, it was not cytotoxic in normal fibroblasts, and 4 showed cytotoxicity values comparable with those displayed by 8 and **CDDP** (Table 1). The cytotoxicity results showed the advantage of introducing the glycine unit into the target molecule 8.

**Table 1:** Cytotoxicity (IC_50_ ± SD [μM]) of compounds 1, 2, 4, 6, 8 and **CDDP** in two cancer cell lines and normal cells after 72 h of exposure

Compound	Cytotoxicity (IC_50_ ± SD [μM])		
	HeLa^a^	MCF7^b^	BJ^c^
1	>50	>50	>50
2	14.6 ± 1.8	16.9 ± 1.3	>50
4	3.8 ± 0.1	5.1 ± 0.8	9.9 ± 2.4
6	10.1 ± 2.3	33.0 ± 0.0^d^	11.9 ± 2.3
8	2.5 ± 0.1	3.3 ± 0.6	3.7 ± 1.0
**CDDP**	11.4 ± 3.8	7.7 ± 1.7	6.9 ± 0.9

a HeLa, cervical cancer.

b MCF7, breast adenocarcinoma.

c BJ, normal human fibroblasts.

d IC_50_ = 27.1 ± 6.0 μM if all three repetitions of the experiment with the MCF7 cancer cells are considered (*cf.* SI, Table S1).

### 2.4. Relationship between nanoscale assembly and cytotoxicity *in vitro*

Finally, a relationship between the nanoscale assembly and cytotoxicity of compounds 6 and 8 was identified using 6 in the MCF7 cancer cell line (SI, Table S1). A difference in the observed cytotoxicity in replication 1 of the experiment (IC_50_ = 15.2 μM) in comparison with the cytotoxicity in replications 2 and 3 of the experiment (IC_50_ = 33.0 μM and IC_50_ = 33.1 μM, respectively) was observed at weekly intervals. This supports our hypothesis that the morphology of the nanoscale assemblies changed during a single replication of the experiment, *i.e.*, in the period between the replications 1 and 2 of the experiment. The conditions during the TEM imaging and cytotoxicity assays were different. A longer time period is applied for cytotoxicity assays between the application of the samples to the cells and the monitoring of the biological experiment (72 h) than during the TEM imaging, which was monitored at a time of 1 h and at a time of 48 h since the sample dissolution. When comparing the micrographs of 6 obtained from the water solution recorded at 1 h (Fig. 1c) and 48 h (Fig. 1d), the development of nanofibers is clearly visible, and this may serve as indirect evidence that the hypothesis is correct. Nevertheless, it is necessary to stress that the nanoscale assembly at the cell wall may be different. However, nanoscale assembly may enable, facilitate or reduce the penetration of the nanostructures formed from the potentially biologically active compounds into the cells, resulting in cytotoxicity found experimentally. Small variations in the IC_50_ values are observed in the HeLa and BJ cells as well; however, the variations in the IC_50_ values are too small to make any conclusion on their basis. In turn, compound 8 displayed higher cytotoxicity than 6, which made tracing the irregularities in the observed cytotoxicity values of 8 much more difficult. Nevertheless, in the past, when other triterpenoid derivatives were studied, we observed variable cytotoxicity, which indicated a possible effect of the nanoscale assembly on the cytotoxicity of the studied compounds that time.^9^ Therefore, the observation made with 6 when tested in the MCF7 cancer cells seems not to be stray and out of the ordinary behaviour. Additional and preliminary information on the extra reference compound S1 can be found in the SI, Tables S2 and S3 and Fig. S1 and S2.

## 3. Conclusions

The nanoscale assembly and cytotoxicity of a series of piperazine amides of betulinic acid, partly decorated with a glycine motif, were studied. The TEM imaging of the nanoscale assemblies of 6 and 8 in methanol and water resulted in several observations. In methanol, compound 6 formed diffuse, cloud-like nanoscale assemblies and isometric nanoparticles that were later transformed into a mixture of isometric assemblies with long fibrous nanoscale structures. In water, a mixture of the cloud-like nanoscale assemblies with long fibrous nanoscale structures appeared at the time of 1 h, while nice nanoscale fibres appeared at the time of 48 h. Compound 8 formed a mixture of the cloud-like nanoscale assemblies and fibres at the time of 1 h, and this morphology changed only slightly with time. In water, compound 8 formed an analogous image as in methanol at the time of 1 h, which also contained isometric nanoparticles of sizes of ∼200 nm. The morphology changed with time to much smaller isometric nanoparticles (20–40 nm) connected by less intensively visible long fibrous nanoscale structures. In cytotoxicity studies, compound 8 was the most active in all tested cells.

## 4. Experimental

### 4.1. General

The NMR measurements were performed on a Bruker AVANCE II 600 MHz spectrometer equipped with a 5 mm TCI cryoprobe in a 5 mm tube in different solvents. The ^1^H NMR and ^13^C NMR spectra were recorded at 600.13 MHz and 150.90 MHz, respectively, in CDCl_3_ (at 293 K) or in DMSO-*d*_6_ (at 373 K) using tetramethylsilane (*δ* = 0.0 – CDCl_3_) or the signal of the solvent (*δ* = 2.50 or 39.50 for ^1^H/^13^C – DMSO-*d*_6_) as internal references. The ^1^H NMR data are presented in the following order: chemical shift (*δ*) expressed in ppm, multiplicity (s, singlet; d, doublet; t, triplet; q, quartet; m, multiplet), number of protons, coupling constants in Hertz. For the unambiguous assignment of both ^1^H and ^13^C signals, 2D NMR ^1^H,^13^C gHSQC and gHMBC spectra were measured using standard parameter sets and pulse programs delivered by the producer of the spectrometer. Infrared spectra (IR) were measured using a Nicolet iS5 FT-IR spectrometer. Mass spectra (MS) were measured with a Waters ZMD mass spectrometer, mostly in a positive ESI mode (coin voltage, CV = 10 to 20 eV). Transmission electron microscopy (TEM) was performed with a Tecnai G2 Spirit Twin 12 microscope (FEI, Brno, Czech Republic). TLC was carried out on silica gel plates (Merck 60F_254_), and the visualization was performed by both UV detection and spraying using a methanolic solution of phosphomolybdic acid (5%), followed by heating. For column chromatography, silica gel 60 (0.063–0.200 mm) from Merck was used. All chemicals and solvents of analytical grade were purchased from regular commercial sources, and the solvents were purified by general methods before use. Betulinic acid were purchased from Dr. Jan Šarek – Betulinines (https://www.betulinines.com). The scanned ^1^H and ^13^C NMR spectra of the prepared compounds, and the calculated ^1^H and ^13^C NMR spectra (ChemBioDraw Ultra, version 12.0) are presented in the SI (Fig. S3–S9).

### 4.2. Synthetic procedures

#### 4.2.1. (3β)-3-(Acetyloxy)lup-20(29)-en-28-oic acid (2)

Acetic anhydride (0.6 mL; 6.35 mmol) was added to a solution of 1 (2.02 g; 4.33 mmol), DIPEA (2 mL; 11.6 mmol) and DMAP (80 mg; 0.66 mmol) in dry THF (10 mL) under an argon atmosphere. The mixture was heated to 95 °C for 3 h. After cooling the mixture to r.t., water was added and the mixture was allowed to stand overnight at r.t., which was later extracted with chloroform. After the extract was dried over sodium sulphate, the solid was filtered off, the solvent was evaporated under reduced pressure, and the residue was purified by column chromatography on silica gel using a chloroform/methanol mixture (100 : 1 to 50 : 1) as the mobile phase, yielding 2 (2.09 g) in 95% yield. ^1^H-NMR (600.13 MHz, CDCl_3_): *δ* [ppm] 0.83 (s, 3H, 23-CH_3_), 0.84 (s, 3H, 24-CH_3_), 0.85 (d, 3H, *J* = 0.6 Hz, 25-CH_3_), 0.93 (s, 3H, 26-CH_3_), 0.97 (d, 3H, *J* = 0.6 Hz, 27-CH_3_), 1.69 (dd, 3H, *J*_1_ = 0.6 Hz, *J*_2_ = 1.4 Hz, 30-CH_3_), 2.04 (s, 3H, CH_3_CO), 3.00 (dt, 1H, *J*_1_ = 4.8 Hz, *J*_2_ = 10.8 Hz, *J*_3_ = 10.8 Hz, 19-CH), 4.46–4.49 (m, 1H, 3-CH), 4.60–4.62 (m, 1H, 29-CH), 4.73–4.75 (m, 1H, 29-CH). ^13^C-NMR (150.92 MHz, CDCl_3_): *δ* [ppm] 14.66 (C27), 16.05 (C26), 16.17 (C25), 16.46 (C24), 18.17(C6), 19.35 (C11), 20.87 (C30), 21.29 (CH_3_CO), 23.70 (C2), 25.46 (C12), 27.95 (C23), 29.70 (C21), 30.58 (C15), 32.16 (C16), 37.04 (C22), 37.14 (C10), 37.26 (C7), 37.81 (C4), 38.40 (C13), 38.44 (C1), 40.72 (C8), 42.44 (C14), 46.95 (C18), 49.30 (C19), 50.42 (C9), 55.44 (C5), 56.40 (C17), 80.96 (C3), 109.73 (C29), 150.36 (C20), 171.03 (CH_3_CO), 181.86 (C28). MS: *m/z* = 497.3 [M–H]^−^ (ESI^−^). For C_32_H_50_O_4_ (498.74), calcd.: C 77.06, H 10.10, found: C 77.10, H 10.08.

#### 4.2.2. *tert*-Butyl 4-[(3β)-3-acetyloxy-28-oxolup-20(29)-en-28-yl]piperazin-1-carboxylate (3)

Oxalyl chloride (4.5 mL; 9.0 mmol) was added to a solution of 2 (0.65 g; 1.29 mmol) in dry DCM (15 mL) under an argon atmosphere, and the mixture was stirred at r.t. for 3 h. The solvent was evaporated under reduced pressure, and the crude product was dissolved in dry DCM (12 mL). The Boc-protected piperazine (0.29 g; 1.55 mmol) and DIPEA (0.58 mL; 3.30 mmol) were added to the above solution, and the resulting mixture was stirred overnight at r.t. The solvent was evaporated under reduced pressure, and the residue was purified by column chromatography on silica gel using a chloroform/methanol (100 : 1) mixture as the mobile phase, yielding 3 (0.681 g) in 84% yield. ^1^H NMR (600.13 MHz, CDCl_3_): *δ* [ppm] 0,78 (1H, dd, *J* = 2.1; 11.2 Hz, H5), 0.82 (3H, s, H25), 0.83 (3H, s, H26), 0.84 (3H, s, H24), 0.92 (3H, s, H23), 0.95 (3H, s, H27), 1.15 (2H, dt, *J* = 3.4; 4.4; 13.4 Hz, H21), 1.46 (9H, s, H7′), 1.68 (3H, dd, *J* = 0.7; 1.3 Hz, H29), 1.80–1.87 (2H, m, H15), 1.91–1.96 (2H, m, H7), 2.03 (3H, s, H2′), 2.07 (2H, dt, *J* = 3.2; 3.2; 13.3 Hz, H16), 2.85 (1H, ddd, *J* = 3.5; 11.4; 13.1 Hz, H13), 2.97 (1H, dt, *J* = 4.2; 11.2; 11.2 Hz, H19), 3.35–3.65 (8H, m, H3′–4′H), 4.46 (1H, dd, *J* = 5.6; 10.7 Hz, H3), 4.58 (1H, dq, *J* = 3 × 1.3; 2.4 Hz, H30), 4.72 (1H, dq, *J* = 3 × 0.7; 2.4 Hz, H30). ^13^C NMR (150.92 MHz, CDCl_3_): *δ* [ppm] 14.6 (q, C27), 16.1 (q, C24), 16.2 (q, C25), 16.5 (q, C26), 18.2 (t, C6), 19.6 (q, C29), 21.1 (t, C11), 21.3 (q, C2′), 23.7 (t, C2), 25.6 (t, C12), 27.9 (q, C23), 28.4 (q, C7′), 29.8 (t, C21), 31.0 (d, C3), 31.3 (t, C15), 32.6 (t, C16), 34.3 (t, C22), 36.0 (s, C10), 36.9 (t, C7), 37.1 (d, C13), 37.8 (s, C4), 38.4 (t, C1), 40.7 (s, C8), 41.9 (s, C14), 43.4 (t, C3′), 44.2 (t, C4′), 45.7 (d, C19), 50.7 (d, C9), 52.7 (d, C18), 54.6 (s, C17), 55.5 (d, C5), 80.2 (s, C6′), 109.3 (t, C30), 151.5 (s, C20), 154.7 (s, C5′), 171.0 (s, C1′), 173.8 (s, C28). MS (ESI^+^, HCOOH): *m/z* = 667.5 [M + H]^+^.

#### 4.2.3. (3β)-28-Oxo-28-(piperazin-1-yl)lup-20(29)en-3-yl acetate (4)

A solution of HCl (gas; 4 M; 4 mL; 16 mmol) in 1,4-dioxane was added to a solution of 3 (0.4 g; 0.60 mmol) in 1,4-dioxane (12 mL), and the mixture was stirred overnight at r.t. The solvent was evaporated under reduced pressure, and the residue was purified by column chromatography on silica gel using a chloroform/methanol (10 : 1 to 1 : 1) mixture as the mobile phase, yielding 4 (0.3 g) in 97% yield. ^1^H NMR (600.13 MHz, CDCl_3_): *δ* [ppm] 0.78 (1H, dd, *J*_1_ = 2.1 Hz, *J*_2_ = 11.2 Hz, H-5), 0.82 (3H, s, H-25), 0.83 (3H, s, H-26), 0.84 (3H, s, H-24), 0.92 (3H, s, H-23), 0.95 (3H, s, H-27), 1.15 (2H, dt, *J*_1_ = 3.4 Hz, *J*_2_ = 3.4 Hz, *J*_3_ = 13.4 Hz, H-21), 1.46 (9H, s, H-7′), 1.68 (3H, dd, *J*_1_ = 0.7 Hz, *J*_2_ = 1.3 Hz, H-30), 1.80 (2H, m, H-15), 1.91 (2H, m, H-7), 2.04 (3H, s, H-2′), 2.07 (2H, dt, *J*_1_ = 3.2 Hz, *J*_2_ = 3.2 Hz, *J*_3_ = 13.3 Hz, H-16), 2.85 (1H, ddd, *J*_1_ = 3.5 Hz, *J*_2_ = 11.4 Hz, *J*_3_ = 13.1 Hz, H-13), 2.97 (1H, dt, *J*_1_ = 4.2 Hz, *J*_2_ = 11.2 Hz, *J*_3_ = 11.2 Hz, H-19), 3.35–3.65 (4H, m, H-3′), 3.35–3.66 (4H, m, H-4′), 4.46 (1H, dd, *J*_1_ = 5.6 Hz, *J*_2_ = 10.7 Hz, H-3), 4.58 (1H, dq, *J*_1_ = 1.3 Hz, *J*_2_ = 1.3 Hz, *J*_3_ = 1.3 Hz, *J*_4_ = 2.4 Hz, H-29), 4.72 (1H, dq, *J*_1_ = 0.7 Hz, *J*_2_ = 0.7 Hz, *J*_3_ = 0.7 Hz, *J*_4_ = 2.4 Hz, H-29). ^13^C NMR (150.90 MHz, CDCl_3_): *δ* [ppm] 14.60 (q, C27), 16.10 (q, C24), 16.20 (q, C25), 16.50 (q, C26), 18.20 (t, C6), 19.60 (q, C30), 21.10 (t, C11), 21.30 (q, C2′), 23.70 (t, C2), 25.60 (t, C12), 27.90 (q, C23), 28.40 (q, C7′), 29.80 (t, C21), 31.00 (d, C3), 31.30 (t, C15), 32.60 (t, C16), 34.30 (t, C22), 36.00 (s, C10), 36.90 (t, C7), 37.10 (d, C13), 37.80 (s, C4), 38.40 (t, C1), 40.70 (s, C8), 41.90 (s, C14), 43.40 (t, C3′), 44.20 (t, C4′), 45.70 (d, C19), 50.70 (d, C9), 52.70 (d, C18), 54.60 (s, C17), 55.50 (d, C5), 80.20 (s, C6′), 109.30 (t, C29), 151.20 (s, C20), 154.70 (s, C5′), 171.00 (s, C1′), 173.80 (s, C28). IR (cm^−1^): 3400 (CO-NH, NH_2_), 2969 (CH_2_), 2900 (CH_3_), 1720 (C≡O), 1630 (C≡O), 1470 (CH_2_), 1405 (CO–H), 1392 (CH_2_), 1249 (C–O–C), 1075 (C–C), 1065 (C–O–C), 1045 (C–O–C), 1027 (C–O–C). MS: *m/z* = 567.6 [M + H]^+^ (ESI^+^). For C_36_H_58_N_2_O_3_ (566.44) calcd.: C 76.28, H 10.31, N 4.94, found: C 76.25, H 10.30, N 4.96.

#### 4.2.4. *tert*-Butyl (2-{4-[(3β)-3-acetyloxy-28-oxolup-20(29)-en-28-yl]piperazin-1-yl}-2-oxoethyl)carbamate (5)

*N*-Methyl morpholine (NMM; 0.5 ml, 4.57 mmol), 1-hydroxybenzotriazol (HOBt; 0.217 g, 1.60 mmol) and Boc-glycine (0.250 g, 1.45 mmol) were added to a solution of 4 (0.803 g, 1.11 mmol), and the mixture was stirred at r.t. for 30 min. Then, EDC·HCl (0.392 g, 2.04 mmol) was added, and the resulting mixture was stirred for an additional 24 h. After stopping the reaction, the mixture was washed with brine, and the organic layer was extracted with chloroform. The extract was dried over sodium sulphate. The solid phase was filtered off, and the solvent was evaporated under reduced pressure. The crude residue was purified by column chromatography on silica gel using a chloroform/methanol (100 : 1) mixture as the mobile phase. Product 5 was obtained in 93% yield. ^1^H NMR (600.13 MHz, DMSO-*d*_6_, 373 K): *δ* [ppm] 0.81 (1H, dd, *J* = 2.0; 11.7 Hz, H5), 0.81 (3H, s, H23), 0.81 (3H, s, H26), 0.84 (3H, d, *J* = 0,6 Hz, H25), 0.89 (3H, bs, H24), 0.96 (3H, d, *J* = 0,4 Hz, H27), 1.13 (2H, dt, *J* = 3.3; 3.3; 13.4 Hz, H15), 1.39 (9H, s, *J* = 1.39 Hz, H9′), 1.54 (1H, t, *J* = 11.2 Hz, H18), 1.66 (2H, dd, *J* = 0.7; 1.4 Hz, H29), 1.75 (2H, ddt, *J* = 7.7; 11.2; 11.2; 13.2 Hz, H21), 1.96 (2H, ddd, *J* = 1.2; 7.5; 12.3 Hz, H7), 1.97 (3H, s, H2′), 2.12 (2H, dt, *J* = 3.5; 3.5; 13.7 Hz, H16), 2.88 (1H, ddd, *J* = 3.7; 11.4; 13.1 Hz, H13), 2.88 (1H, dt, *J* = 4.5; 11.0; 11.0 Hz, H19), 3.40 (2H, dt, *J* = 5.0; 5.0; 13.4 Hz, H4′), 3.43 (2H, dt, *J* = 5.2; 5.2; 13.4 Hz, H4′), 3.52 (4H, t, *J* = 5.1 Hz, H3′), 3.81 (2H, d, *J* = 5.6 Hz, H6′), 4.38 (1H, dd, *J* = 5.0; 11.3 Hz, H3), 4.56 (1H, dq, *J* = 3 × 1.4; 2.4 Hz, H30), 4.67 (1H, dq, *J* = 3 × 0.7; 2.4 Hz, H30). ^13^C NMR (150.92 MHz, DMSO-*d*_6_, 373 K): *δ* [ppm] 13.8 (q, C27), 15.3 (q, C24), 15.4 (q, C25), 15.8 (q, C26), 17.3 (t, C6), 18.7 (q, C29), 20.2 (q, C2′), 20.4 (t, C11), 22.9 (t, C2), 24.9 (t, C12), 27.2 (q, C23), 27.7 (q, C9′), 28.9 (t, C15), 30.5 (t, C21), 31.4 (t, C16), 33.5 (t, C22), 34.7 (t, C7), 36.0 (d, C13), 36.4 (s, C10), 37.0 (s, C4), 37.5 (t, C1), 41.2 (s, C8), 41.5 (t, C4′), 42.4 (t, C3′), 44.0 (t, C6′), 45.0 (s, C14), 45.0 (d, C19), 49.8 (d, C9), 52.0 (d, C18), 53.8 (s, C17), 54.5 (d, C5), 77.6 (s, C8′), 79.7 (d, C3), 108.3 (t, C30), 150.5 (s, C20), 154.9 (s, C7′), 167.2 (s, C5′), 169.3 (s, C1′), 172.7 (s, C28). IR (cm^−1^): *ν̃* = 3419, 3324 (br, w), 2940 (m), 2865 (w-m), 1718 (s), 1636 (s), 1457(m), 1406 (m), 1391 (m), 1365 (m-s), 1244 (vs), 1186 (m), 1165 (s), 1011 (s), 978 (m), 750 (s).

#### 4.2.5. (3β)-28-(4-Glycylpiperazin-1-yl)-28-oxolup-20(29)-en-3-yl acetate (6)

Compound 6 was prepared using the procedure described for the synthesis of 4 (section 4.2.3.) in 95% yield.

^1^H NMR (600.13 MHz, DMSO-*d*_6_, 373 K): *δ* [ppm] 0.83 (1H, dd, *J* = 1.7; 11.6 Hz, H5), 0.83 (3H, s, H23), 0.83 (3H, s, H26), 0.86 (3H, d, *J* = 0.6 Hz, H25), 0.91 (3H, s, H24), 0.97 (3H, d, *J* = 0.4 Hz, H27), 1.14 (2H, dt, *J* = 3.4; 3.4; 13.4 Hz, H15), 1.19 (2H, dq, *J* = 4.6; 3 × 12.8 Hz, H11), 1.56 (1H, t, *J* = 11.3 Hz, H18), 1.68 (3H, dd, *J* = 0.7; 1.4 Hz, H29), 1.76 (2H, ddt, *J* = 7.7; 11.2; 11.2; 13.3 Hz, H21), 1.97 (2H, ddd, *J* = 1.2; 7.7; 12.1 Hz, H7), 1.99 (3H, s, H2′), 2.12 (2H, dt, *J* = 3.6; 3.6; 13.7 Hz, H16), 2.88 (1H, ddd, *J* = 3.7; 11.3; 13.0 Hz, H13), 2.88 (1H, dt, *J* = 4.5; 10.9; 10.9 Hz, H19), 3.39–3.47 (4H, m, H4′), 3.51 (2H, s, H6′), 3.55 (4H, t, *J* = 5.5 Hz, H3′), 4.40 (1H, dd, *J* = 5.1; 11.4 Hz, H3), 4.57 (1H, dq, *J* = 3 × 1.4; 2.4 Hz, H30), 4.69 (1H, dq, *J* = 3 × 0.7; 2.4 Hz, H30). ^13^C NMR (150.92 MHz, DMSO-*d*_6_, 373 K): *δ* [ppm] 13.8 (q, C27), 15.3 (q, C24), 15.4 (q, C25), 15.8 (q, C26), 17.3 (t, C6), 18.7 (q, C29), 20.1 (t, C11), 20.2 (q, C2′), 22.9 (t, C2), 24.9 (t, C12), 27.2 (q, C23), 28.9 (t, C15), 30.5 (t, C21), 31.4 (t, C16), 33.5 (t, C22), 34.7 (t, C7), 36.0 (s, C10), 36.4 (d, C13), 37.0 (s, C4), 37.5 (t, C1), 41.2 (s, C8), 41.2 (t, C6′), 43.9 (t, C3′), 43.9 (t, C4′), 45.0 (d, C19), 45.1 (s, C14), 49.7 (d, C9), 52.0 (d, C18), 53.8 (s, C17), 54.5 (d, C5), 79.7 (d, C3), 108.3 (t, C30), 150.5 (s, C20), 169.2 (s, C5′), 169.3 (s, C1′), 172.7 (s, C28). IR (cm^−1^): *ν̃* = 3400 (br, w), 2940 (s), 2865 (m), 1729 (m), 1634 (s), 1453 (m), 1404 (m), 1370 (m), 1245 (vs), 1186 (m), 1009 (s), 978 (m), 750 (s). MS (ESI^+^, CH_3_COOH): *m/z* = 624.6 [M + H]^+^. For C_38_H_61_N_3_O_4_ (623.91) calcd:. C 73.15, H 9.85, N 6.73, found: C 73.18, H 9.83, N 6.75.

#### 4.2.6. *tert*-Butyl (2-{4-[(3β)-3-hydroxy-28-oxolup-20(29)-en-28-yl]piperazin-1-yl}-2-oxoethyl)carbamate (7)

A solution of lithium hydroxide hydrate (0.116 g, 2.77 mmol) in methanol (20 mL) was added to a solution of 5 in methanol (5 mL), and the reaction mixture was heated to reflux for 4 h. The solvent was evaporated under reduced pressure, and the crude residue was purified by column chromatography on silica gel using a chloroform/methanol (100 : 1) mixture as the mobile phase, affording 7 in 86% yield. ^1^H NMR (600.13 MHz, DMSO-*d*_6_, 373 K): *δ* [ppm] 0.66 (1H, dd, *J* = 1.9; 11.2 Hz, H5), 0.69 (3H, s, H25), 0.81 (3H, d, *J* = 0.7 Hz, H26), 0.89 (3H, s, H24), 0.90 (3H, s, H23), 0.94 (3H, dd, *J* = 0.7; 1.4 Hz, H30), 1.12 (2H, dt, *J* = 3.4; 3.4; 13.4 Hz, H15), 1.16 (2H, dq, *J* = 4.6; 12.8; 12.8; 12.8 Hz, H11), 1.40 (9H, s, H7′), 1.52 (1H, t, *J* = 11.2 Hz, H18), 1.59 (2H, dt, *J* = 3.6; 3.6; 12.9 Hz, H1), 1.60–1.65 (2H, m, H12), 1.73 (2H, ddt, *J* = 7.9; 11.2; 11.2; 13.3 Hz, H21), 1.96 (2H, ddd, *J* = 1.2; 7.7; 11.9 Hz, H7), 2.13 (2H, dt, *J* = 3.5; 3.5; 13.5 Hz, H16), 2.66 (1H, dt, *J* = 5.0; 5.0; 12.1 Hz, H4′), 2.68 (1H, dt, *J* = 5.0; 5.0; 12.1 Hz, H4′), 2.89 (1H, dt, *J* = 4.5; 10.9; 10.9 Hz, H19), 2.91 (1H, ddd, *J* = 3.6; 11.4; 13.2 Hz, H13), 3.01 (1H, dd, *J* = 6.5; 10.3 Hz, H3), 3.40–3.42 (4H, m, H2′), 3.44–3.47 (4H, m, H1′), 4.56 (1H, dq, *J* = 1.4; 1.4; 1.4; 2.4 Hz, H29), 4.67 (1H, dq, *J* = 0.7; 0.7; 0.7; 2.4 Hz, H29). ^13^C NMR (150.92 MHz, DMSO-*d*_6_, 373 K): *δ* [ppm] 13.9 (q, C27), 15.1 (q, C25), 15.4 (q, C24), 15.4 (q, C26), 17.6 (t, C6), 18.8 (q, C30), 26.8 (t, C2), 27.6 (q, C23), 27.8 (q, C7′), 28.9 (t, C15), 30.6 (t, C21), 31.4 (t, C16), 33.7 (t, C22), 34.7 (t, C7), 36.0 (d, C13), 38.1 (t, C1), 38.1 (s, C4), 41.2 (s, C8), 44.0 (t, C2′), 45.1 (t, C4′), 45.2 (s, C14), 45.2 (d, C19), 45.5 (t, C1′), 50.0 (d, C9), 52.1 (d, C18), 53.8 (s, C17), 54.8 (d, C5), 76.6 (d, C3), 77.1 (s, C6′), 108.3 (t, C29), 150.6 (s, C20), 154.7 (s, C5′), 171.7 (s, C3′), 172.2 (s, C28). IR (cm^−1^): *ν̃* = 3376 (br), 2937 (m), 2865 (w), 1693 (m), 1633 (s), 1587 (s), 1446 (s), 1392 (s), 1367 (m), 1255 (w), 1168 (m), 1029 (m).

#### 4.2.7. (3β)-28-[4-(Aminoacetyl)piperazin-1-yl]-3-hydroxylup-20(29)-en-28-one (8)

Compound 8 was prepared using the procedure described for the synthesis of 4 (section 4.2.3.) in 91% yield. ^1^H NMR (600.13 MHz, DMSO-*d*_6_, 373 K): *δ* [ppm] 0.68 (1H, dd, *J* = 1.9; 11.0 Hz, H5), 0.69 (3H, s, H25), 0.81 (3H, d, *J* = 0.7 Hz, H26), 0.90 (3H, s, H24), 0.91 (3H, s, H23), 0.96 (3H, s, H27), 0.97 (1H, dq, *J* = 4.4; 3 × 12.8 Hz, H12), 1.14 (2H, dt, *J* = 3.4; 3.4; 13.3 Hz, H15), 1.18 (2H, dq, *J* = 4.6; 3 × 12.9 Hz, H11), 1.28 (1H, dd, *J* = 3.1; 12.4 Hz, H9), 1.55 (1H, t, *J* = 11.1 Hz, H18), 1.60 (2H, dt, *J* = 3.6; 3.6; 13.0 Hz, H1), 1.64 (1H, ddt, *J* = 3.0; 3.0; 4.6; 13.1 Hz, H12), 1.67 (3H, dd, *J* = 0.7; 1.4 Hz, H30), 1.76 (2H, ddt, *J* = 7.7; 11.3; 11.3; 13.3 Hz, H21), 1.97 (2H, ddd, *J* = 1.2; 7.7; 12.4 Hz, H7), 2.13 (2H, dt, *J* = 3.6; 3.6; 13.7 Hz, H16), 2.88 (1H, ddd, *J* = 3.7; 11.6; 13.3 Hz, H13), 2.89 (1H, dt, *J* = 4.6; 11.0; 11.0 Hz, H19), 3.02 (1H, dd, *J* = 6.1; 10.2 Hz, H3), 3.40–3.46 (4H, m, H2′), 3.53–3.56 (4H, m, H1′), 4.52 (2H, s, H4′), 4.57 (1H, dq, *J* = 3 × 1,4; 2,4 Hz, H29), 4.68 (1H, dq, *J* = 3 × 0,7; 2,4 Hz, H29). ^13^C NMR (150.92 MHz, DMSO-*d*_6_, 373 K): *δ* [ppm] 13.9 (q, C27), 15.1 (q, C25), 15.3 (q, C24), 15.4 (q, C26), 17.5 (t, C6), 18.7 (q, C30), 20.4 (t, C11), 24.9 (t, C12), 26.7 (t, C2), 27.6 (q, C23), 28.9 (t, C15), 30.5 (t, C21), 31.4 (t, C16), 33.7 (t, C22), 34.7 (t, C7), 36.0 (d, C13), 36.5 (s, C10), 38.0 (t, C1), 38.0 (s, C4), 41.1 (t, C4′), 41.2 (s, C8), 42.3 (t, C2′), 43.9 (t, C1′), 45.1 (s, C14), 45.1 (d, C19), 50.0 (d, C9), 52.0 (d, C18), 53.8 (s, C17), 54.8 (d, C5), 76.6 (d, C3), 108.3 (t, C29), 150.5 (s, C20), 169.0 (s, C3′), 172.7 (s, C28). IR (cm^−1^): *ν̃* = 3382 (br), 2936 (m), 2864 (m), 1633 (s), 1453 (m), 1403 (m), 1372 (m), 1284 (w), 1250 (w), 1187 (m), 1009 (m). MS (ESI^+^, CH_3_COOH): *m/z* = 582.3 [M + H]^+^. For C_36_H_59_N_3_O_3_ (581.87) calcd.: C 74.31, H 10.22, N 7.22, found: C 74.28, H 10.24, N 7.20.

### 4.3. Transmission electron microscopy

The general procedure for the sample preparation and collection of micrographs was described in full detail recently.^25–27^

### 4.4. Cell cultures and cytotoxicity screening tests

The human cancer cell lines used in this investigation included cervical carcinoma (HeLa) and breast adenocarcinoma (MCF7) from the European Collection of Authenticated Cell Cultures (Salisbury, UK). Human foreskin fibroblasts (BJ) were used as the reference cells (ATCC, Manassas, VA, USA). The process of culturing the cells and their maintenance was also described in full detail recently.^3^ Cytotoxicity screening tests were performed according to the standard experimental procedure using Alamar Blue.^9^

## Author contributions

The manuscript was written through contributions from all the authors. All the authors have given approval to the final version of the manuscript. Conceptualization: Z. W., M. Š., and L. R.; methodology: M. W., and M. K.; investigation: M. W., M. K., and D. Š.; resources: Z. W., L. R., and M. Š.; data curation: M. W., M. Š., and D. Š.; writing – original draft preparation: M. W., M. Š., L. R., D. Š., and Z. W.; writing – review and editing: M. Š., L. R., and Z. W.; project administration: M. Š., L. R., and Z. W.; funding acquisition: M. Š., L. R., and Z. W.

## Conflicts of interest

There are no conflicts to declare.

## Abbreviations

BJ: Normal human fibroblasts
DCM: Dichloromethane
DIPEA: *N*-Ethyl-*N*,*N*-diisopropyl amine
EDC·HCl: *N*-Ethyl-*N′*-(3-dimethylaminopropyl)carbodiimide hydrochloride
HeLa: Cervical cancer cells
HOBt: 1-Hydroxybenzotriazole
MCF7: Breast adenocarcinoma cells
NMM: *N*-Methylmorpholine

## Supplementary Material

MD-017-D6MD00638H-s001

## Data Availability

The data supporting this article have been included as part of the supplementary information (SI). Supplementary information: ^1^H and ^13^C NMR spectra measured on a Bruker AVANCE II 600 MHz spectrometer, calculated NMR spectra using the ChemBioDraw Ultra software, version 12.0, additional details on TEM imaging, and additional details on the cytotoxicity screening tests. See DOI: https://doi.org/10.1039/d6md00638h. Further data that support the findings of this study are available from the corresponding author upon reasonable request.
