# Supplementary material for: Relationship between nanoscale assembly and cytotoxicity in the piperazine amides of betulinic acid: an in vitro study
Source: RSC Med Chem. 2026 Sep 8;17(10):5050–7. doi: 10.1039/d6md00638h (PMC13635896; doi:10.1039/d6md00638h)
Supplement: Supplementary file 1 [file MD-017-D6MD00638H-s001.pdf]

Supplementary Information (SI) for RSC Medicinal Chemistry.
This journal is © The Royal Society of Chemistry

**ELECTRONIC SUPPLEMENTARY INFORMATION (ESI)**

# A relationship between nanoscale assembly and cytotoxicity in the piperazine amides of betulinic acid: An *in vitro* study

Martina Wimmerová,[a] Miroslav Šlouf,[b] Lucie Rárová,[c] Marie Kvasnicová,[c] David Šaman,[d] Zdeněk Wimmer *,[a]

[a] University of Chemistry and Technology in Prague, Department of Chemistry of Natural Compounds, CZ-16628 Prague, Czech Republic.

[b] Institute of Macromolecular Chemistry, Czech Academy of Sciences, CZ-16206 Prague, Czech Republic.

[c] Laboratory of Growth Regulators, Faculty of Science, Palacký University & Institute of Experimental Botany, Czech Academy of Sciences, CZ-77900 Olomouc, Czech Republic.

[d] Institute of Organic Chemistry and Biochemistry, Czech Academy of Sciences, CZ-16610 Prague, Czech Republic.

# 1. Results and discussion

## 1.1. Cytotoxicity and its possible relationships with the nanoscale assembly

A possible relationship between the nanoscale assembly and cytotoxicity of the compounds **6** and **8** seems to be improbable. The differences in the nanoscale assembly of both compounds in the samples made in water are noticeable (cf. Figure 1c *vs.* 1g and Figure 1d *vs.* 1h). Nevertheless, the differences in the nanoscale assembly of **6** and **8** may possibly be responsible for the observed differences in the cytotoxicity data. These differences may more obviously be explained by the different bioavailability of both compounds by the tested cells due to the dynamic nanoscale assembly process, in which the images of the formed nanoscale assemblies change by time. This phenomenon may be documented by the cytotoxicity of **6** found in the three replications in the MCF7 cancer cells ($IC_{50}$ = 15.2 μM in the replication 1 *vs.* $IC_{50}$ = 33.0 μM and $IC_{50}$ = 33.1 μM in the replications 2 and 3). Moreover, the conditions during the TEM imaging and those during the cytotoxicity assays are different, because a longer time is applied since the moment of the application of the samples to the cells and the moment when the biological experiment is terminated (72 h) than during the TEM imaging (1 h and 48 h). In the past, when other triterpenoid derivatives had been studied, we have observed a variable cytotoxicity, which had indicated a possible effect of the nanoscale assembly on the cytotoxicity of the studied compounds that time.[9] However, the studied compounds **6** and **8** showed no irregularities during the replication of the biological experiments in contrast to the earlier experiments mentioned above.[9] Usually three replications are performed with each studied system consisting of the compound and the cancer cell line, unless irregularities appear; then the number of replications may increase to avoid any failing experiment (Table S1).

**Table S1.** Cytotoxicity ($IC_{50}$ [μM]) of the compounds **6** and **8** in two cancer cell lines and normal cells after 72 h of exposure [a]

| Comp. | HeLa ($IC_{50}$ [μM]) | | | MCF7 ($IC_{50}$ [μM]) | | | BJ ($IC_{50}$ [μM]) | | |
|---|---|---|---|---|---|---|---|---|---|
| | Replications of the cytotoxicity assays | | | | | | | | |
| | 1 | 2 | 3 | 1 | 2 | 3 | 1 | 2 | 3 |
| **6** | 8.6 | 12.7 | 8.9 | 15.2 | 33.0 | 33.1 | 12.1 | 14.0 | 9.5 |
| **8** | 2.5 | 2.6 | 2.4 | 3.9 | 2.8 | 3.3 | 3.9 | 2.6 | 4.6 |

[a] Cytotoxicity values ($IC_{50}$ [μM]) achieved in the three replicates of the experiments using the stock solution prepared at the very beginning of the cytotoxicity assays and measured at weekly intervals.

1.2. Preliminary result with the compound S1 to compare the TEM micrographs

**Figure S1**. Structure of the compound **S1**.

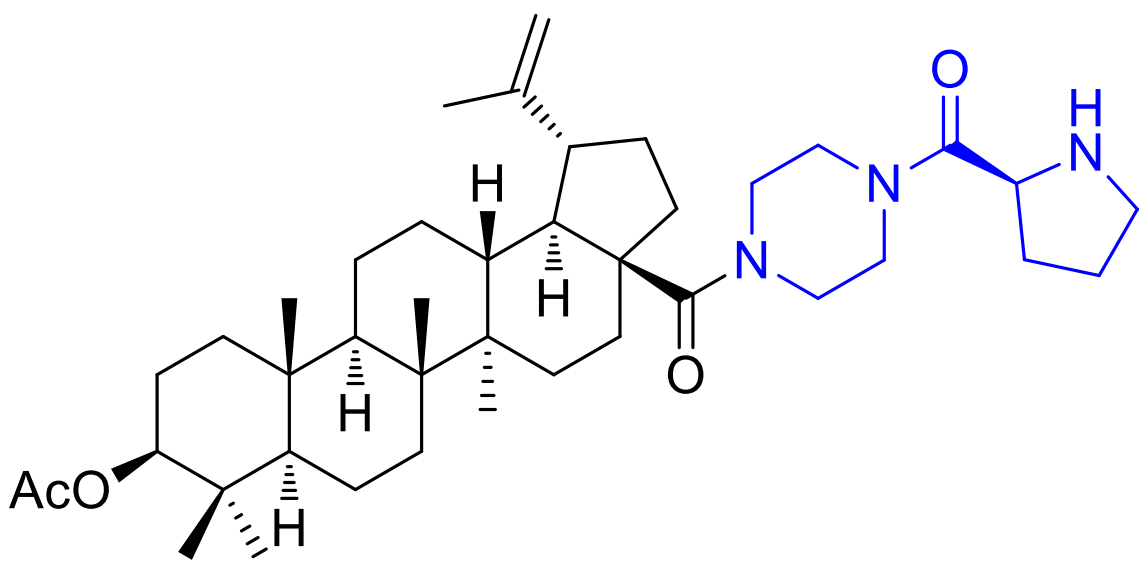


**S1**

When the cytotoxicity of the compounds **1**, **2**, **4**, **6** and **8** was studied, compound **S1** (Figure S1), belonging to the neighboring series of compounds being under investigation at present, was taken as a reference compound. The compound **S1** displayed cytotoxicity similar that of the compound **6** (Tables S2 and S3). Due to that finding, compound **S1** was also subjected to the nanoscale assembly investigation. The obtained micrographs showed certain similarity of

the nanoscale assembling of **S1** to the compound **8**, when its nanostructures were studied in methanol, and to the compound **6**, when its nanostructures were studied in water (Figure S2).

**Table S2**. Cytotoxicity ($IC_{50}$ ± SD [μM]) of the compound **S1** and **CDDP** in two cancer cell lines and normal cells after 72 h of exposure

| Compound | Cytotoxicity ($IC_{50}$ ± SD [μM]) | | |
|---|---|---|---|
| | HeLa [a] | MCF7 [b] | BJ [c] |
| **S1** | 8.3 ± 0.3 | 36.2 ± 2.0 [d] | 13.4 ± 0.6 |
| **CDDP** | 11.4 ± 3.8 | 7.7 ± 1.7 | 6.9 ± 0.9 |

[a] HeLa, cervical cancer; [b] MCF7, breast adenocarcinoma; [c] BJ, normal human fibroblasts; [d] $IC_{50}$ = 28.9 ± 3.0 μM if all three repetitions of the experiment with the MCF7 cancer cells are considered (cf. Table S3).

**Table S3.** Cytotoxicity ($IC_{50}$ [μM]) of the compound **S1** in two cancer cell lines and normal cells after 72 h of exposure [a]

| Comp. | HeLa ($IC_{50}$ [μM]) | | | MCF7 ($IC_{50}$ [μM]) | | | BJ ($IC_{50}$ [μM]) | | |
|---|---|---|---|---|---|---|---|---|---|
| | Replications of the cytotoxicity assays | | | | | | | | |
| | 1 | 2 | 3 | 1 | 2 | 3 | 1 | 2 | 3 |
| **S1** | 8.5 | 8.5 | 7.9 | 14.4 | 37.6 | 34.8 | 13.9 | 13.6 | 12.7 |

[a] Cytotoxicity values ($IC_{50}$ [μM]) achieved in the three replicates of the experiments using the stock solution prepared at the very beginning of the cytotoxicity assays and measured at weekly intervals.

**Figure S2**. Nanoscale assembly of **S1** in methanol and in water: (a) **S1**, methanol, 1 h; (b) **S1**, methanol, 48 h; (c) **S1**, water, 1 h; (d) **S1**, water, 48 h.

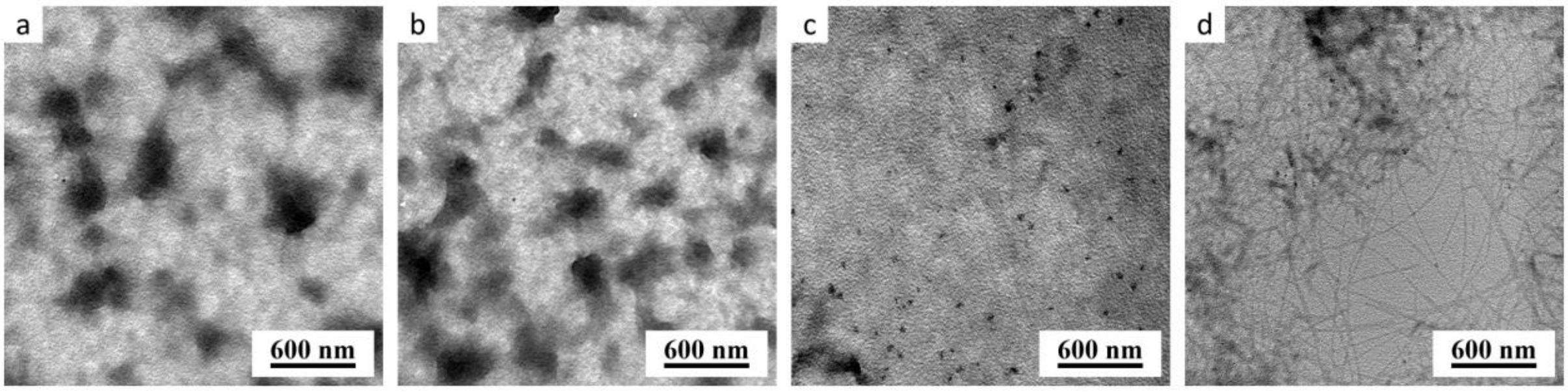


Legend: The time of imaging (1 h and 48 h) was measured from the point of complete dissolution of the compound **S1**.

## 2. Experimental part: NMR spectra (Figures S3–S9)

2.1. (3β)-3-(Acetyloxy)lup-20(29)-en-28-oic acid (**2**) – **Figure S3**

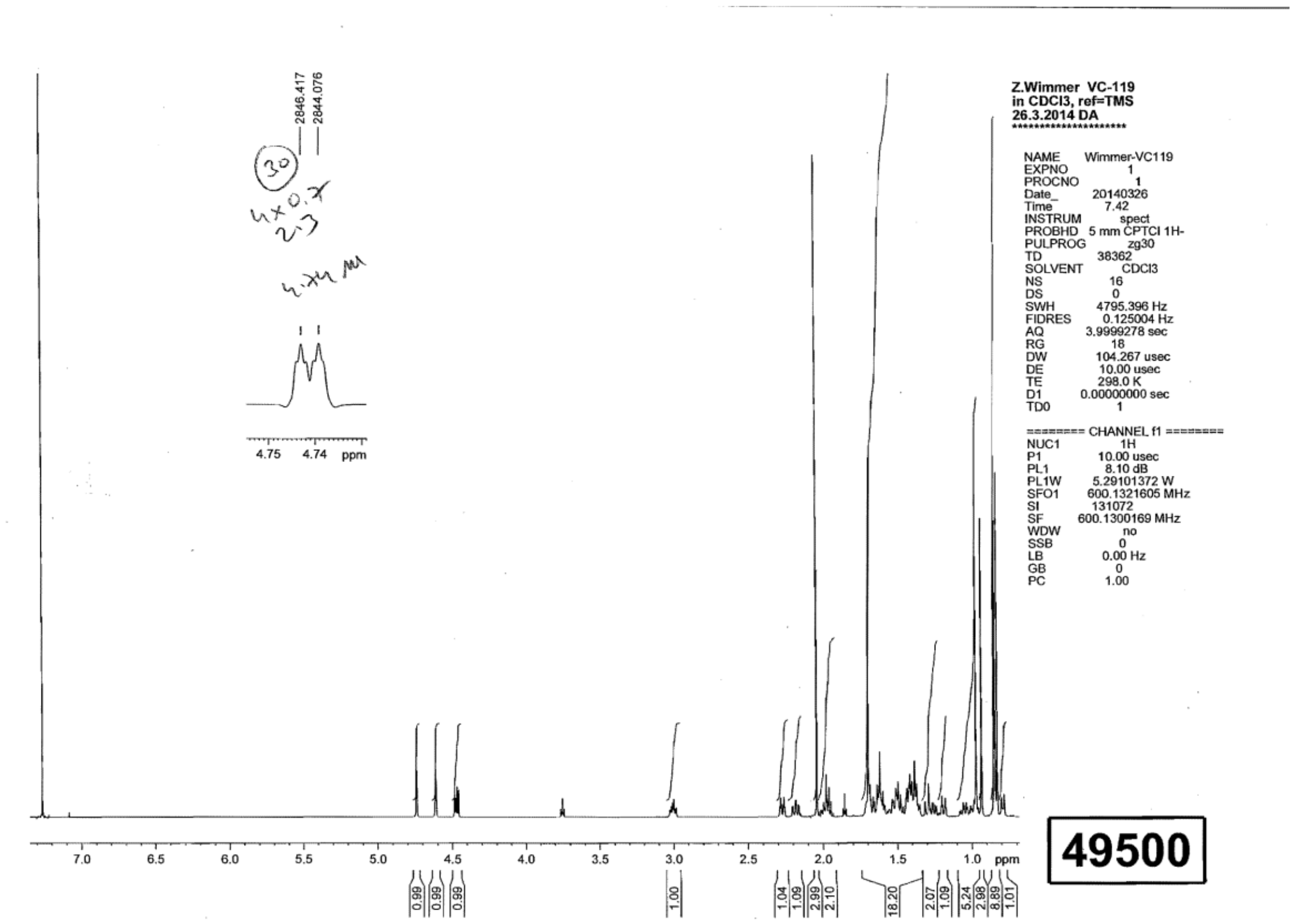


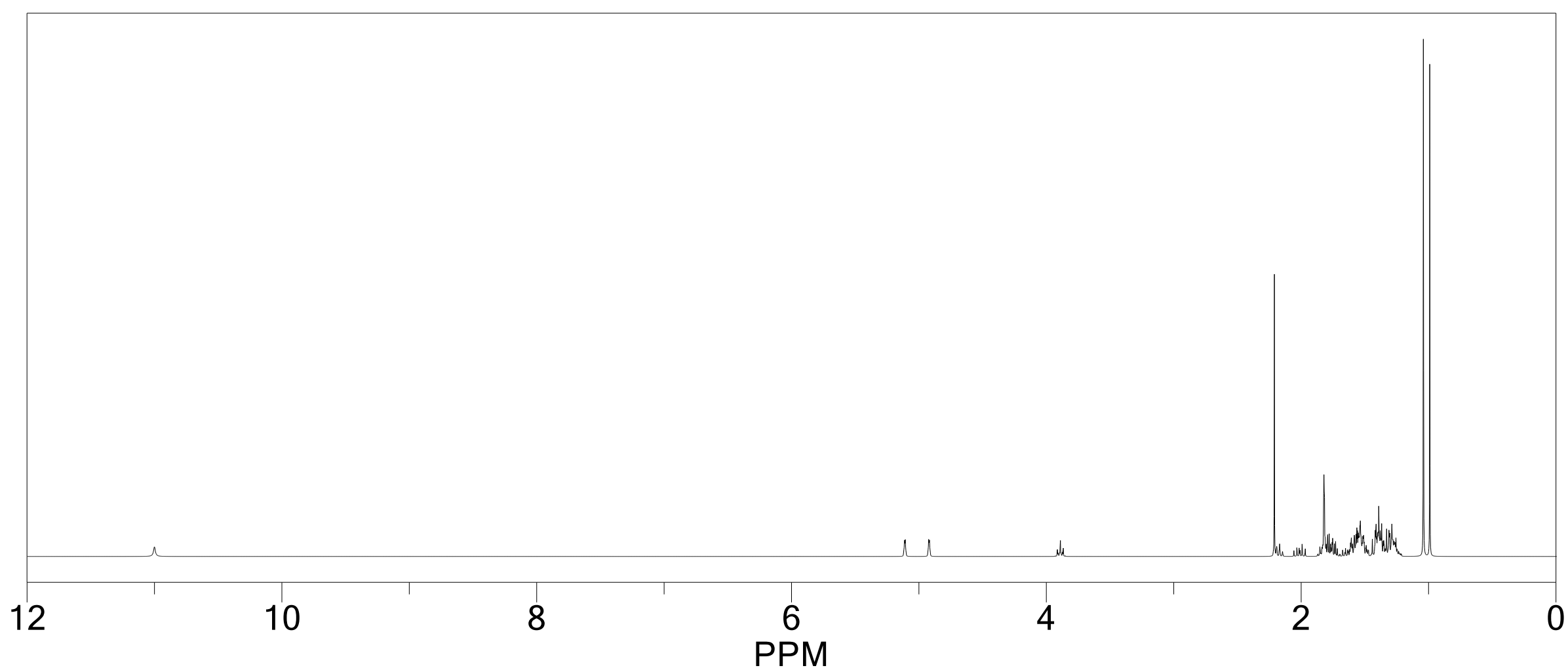

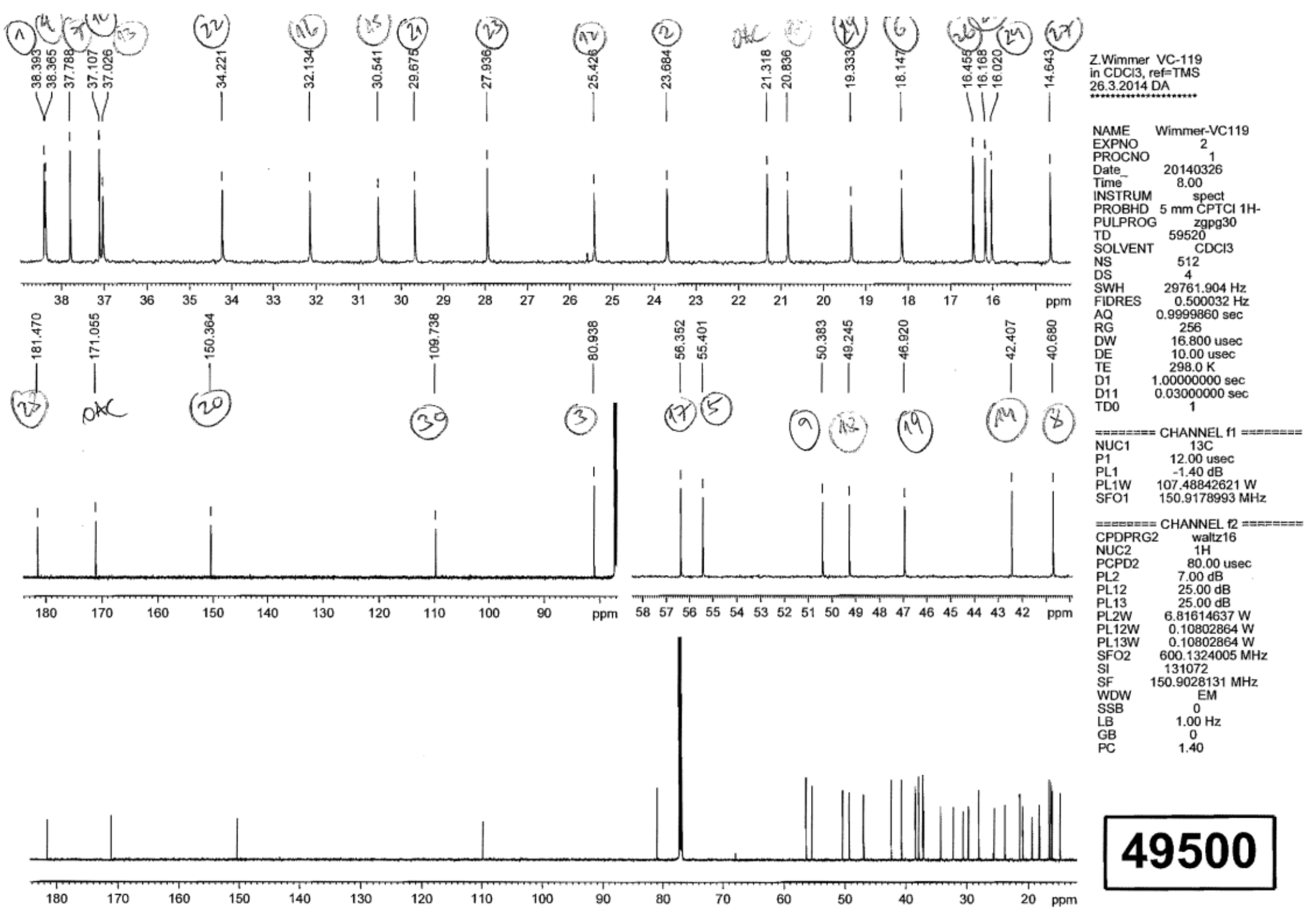
38.393
38.365
37.788
37.107
37.026
34.221
32.134
30.541
29.675
27.936
25.426
23.684
21.318
20.836
19.333
18.147
16.455
16.168
16.020
14.643
ppm
181.470
171.055
150.364
109.738
80.938
56.352
55.401
50.383
49.245
46.920
42.407
40.680
Z.Wimmer VC-119
in CDCl3, ref=TMS
26.3.2014 DA
NAME Wimmer-VC119
EXPNO 2
PROCNO 1
Date_ 20140326
Time 8.00
INSTRUM spect
PROBHD 5 mm CPTCI 1H-
PULPROG zgpg30
TD 59520
SOLVENT CDCl3
NS 512
DS 4
SWH 29761.904 Hz
FIDRES 0.500032 Hz
AQ 0.9999860 sec
RG 256
DW 16.800 usec
DE 10.00 usec
TE 298.0 K
D1 1.00000000 sec
D11 0.03000000 sec
TD0 1
======== CHANNEL f1 ========
NUC1 13C
P1 12.00 usec
PL1 -1.40 dB
PL1W 107.48842621 W
SFO1 150.9178993 MHz
======== CHANNEL f2 ========
CPDPRG2 waltz16
NUC2 1H
PCPD2 80.00 usec
PL2 7.00 dB
PL12 25.00 dB
PL13 25.00 dB
PL2W 6.81614637 W
PL12W 0.10802864 W
PL13W 0.10802864 W
SFO2 600.1324005 MHz
SI 131072
SF 150.9028131 MHz
WDW EM
SSB 0
LB 1.00 Hz
GB 0
PC 1.40
49500

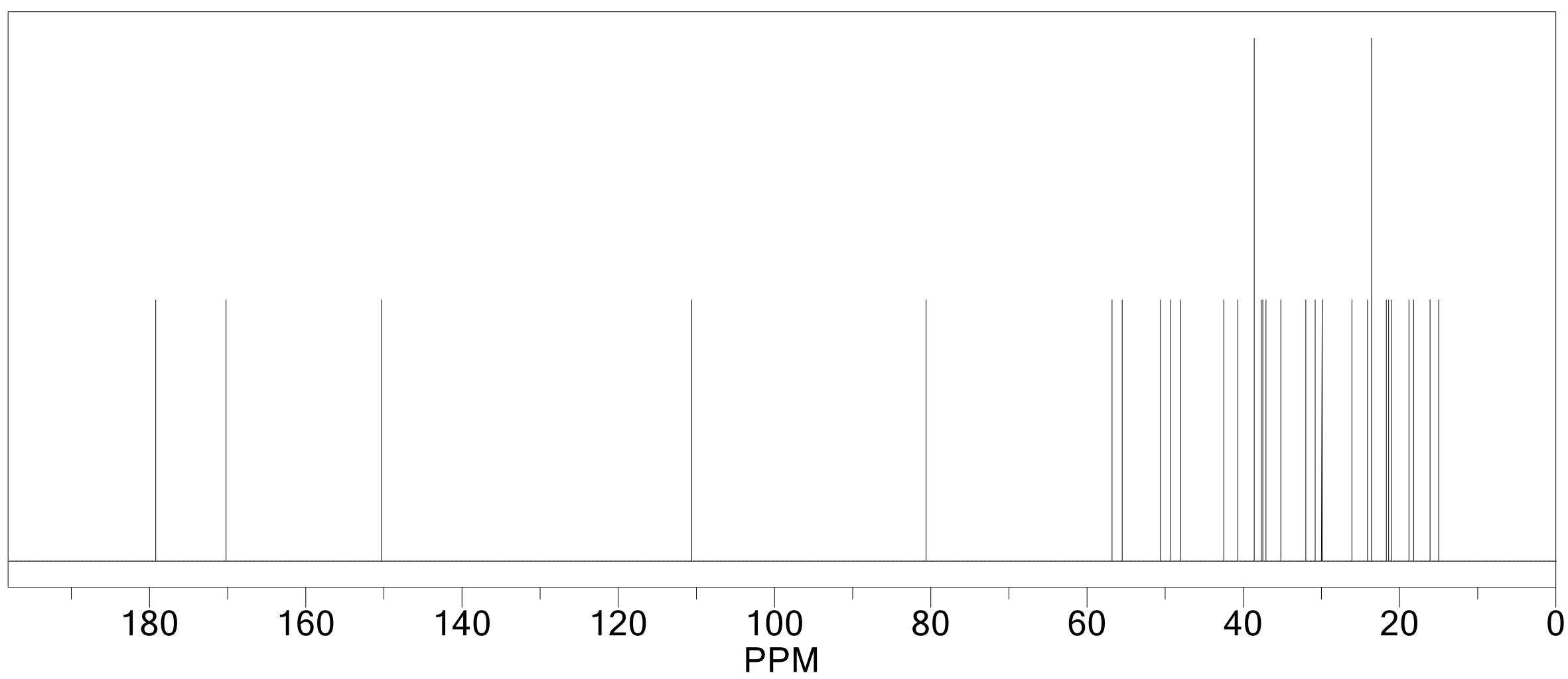
180
160
140
120
100
80
60
40
20
0
PPM

2.2. *tert*-Butyl 4-[(3β)-3-acetyloxy-28-oxolup-20(29)-en-28-yl]piperazin-1-carboxylate (**3**)

**Figure S4**

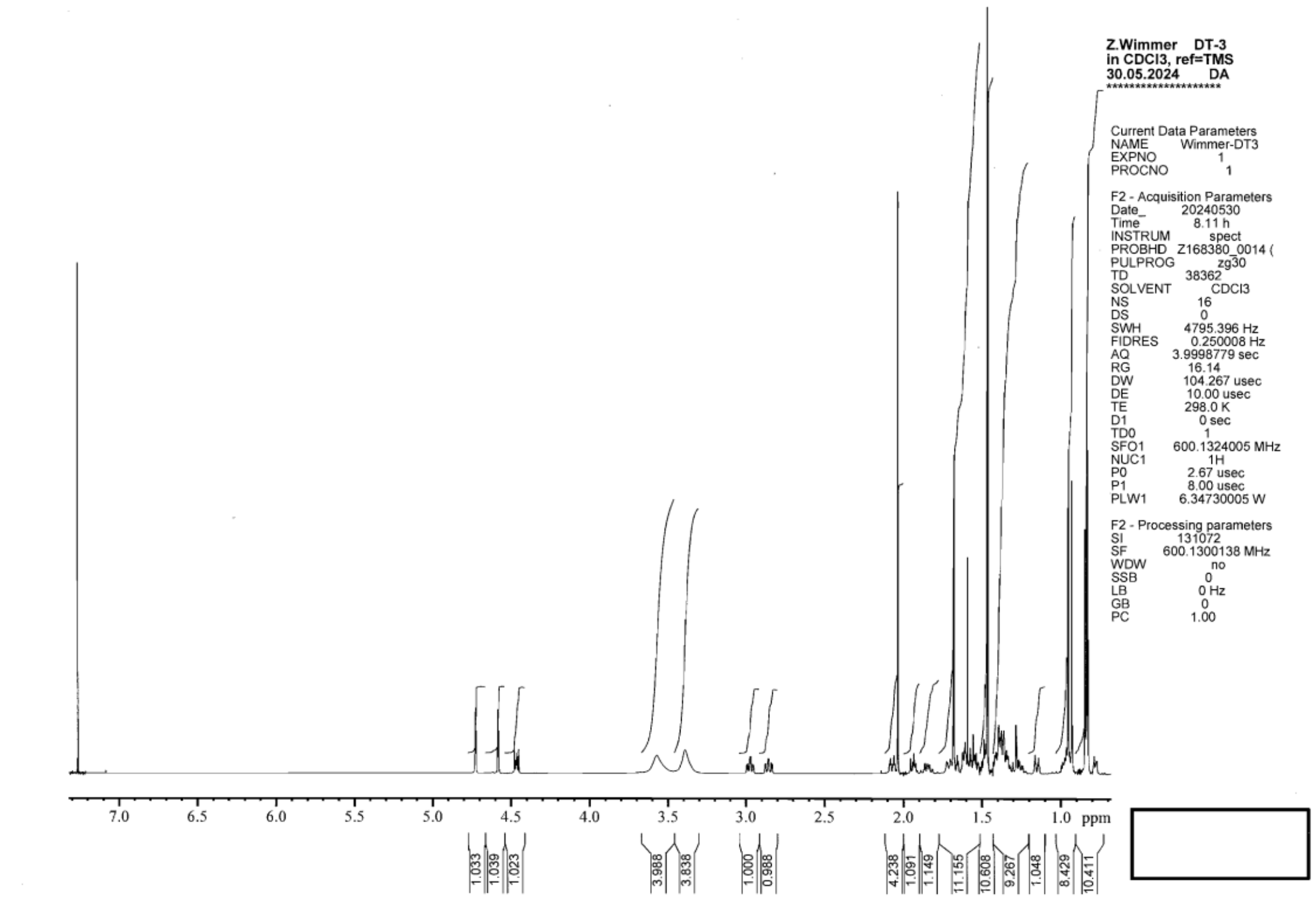


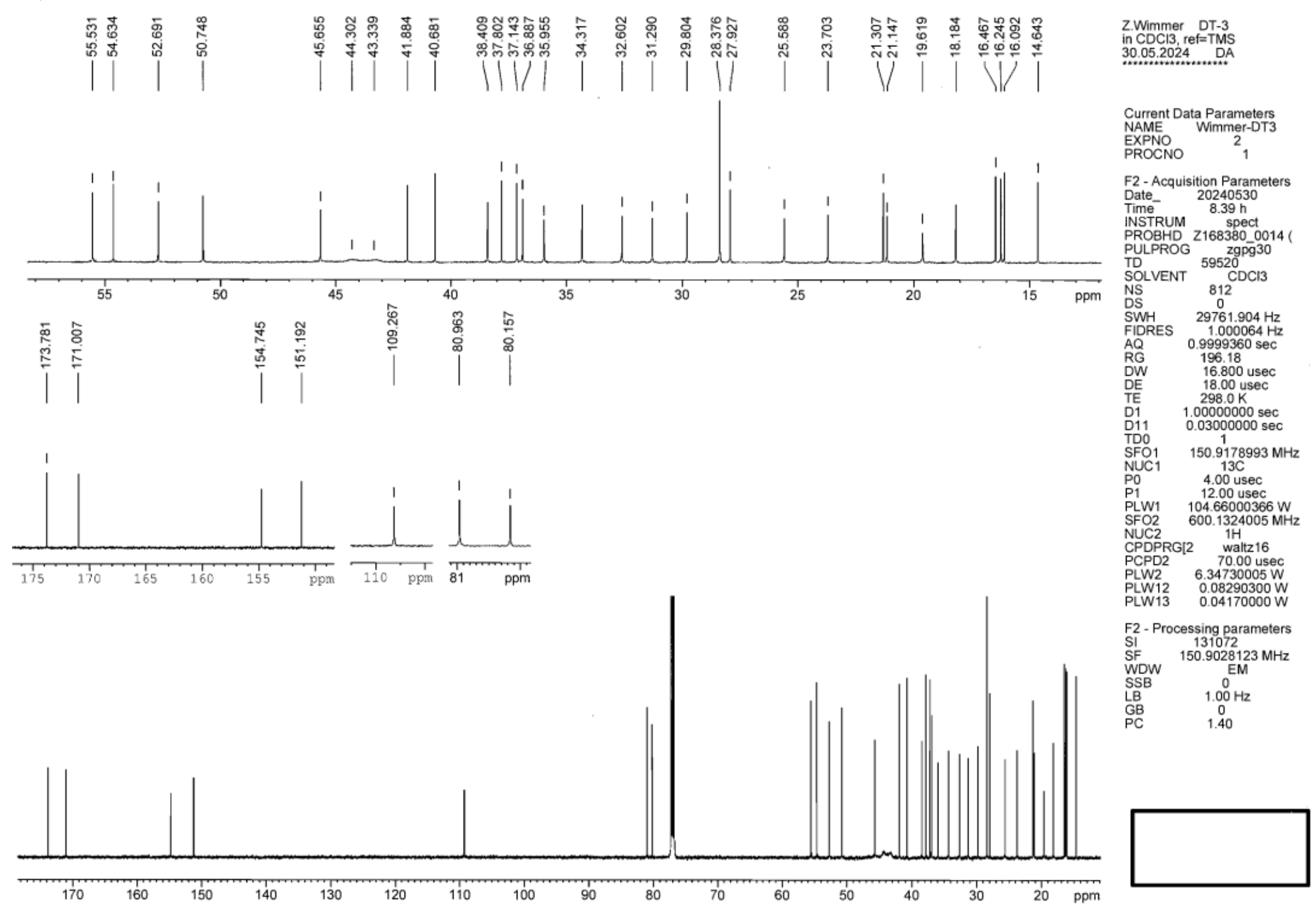

2.3. (3β)-28-Oxo-28-(piperazin-1-yl)lup-20(29)en-3-yl acetate (**4**) – **Figure S5**

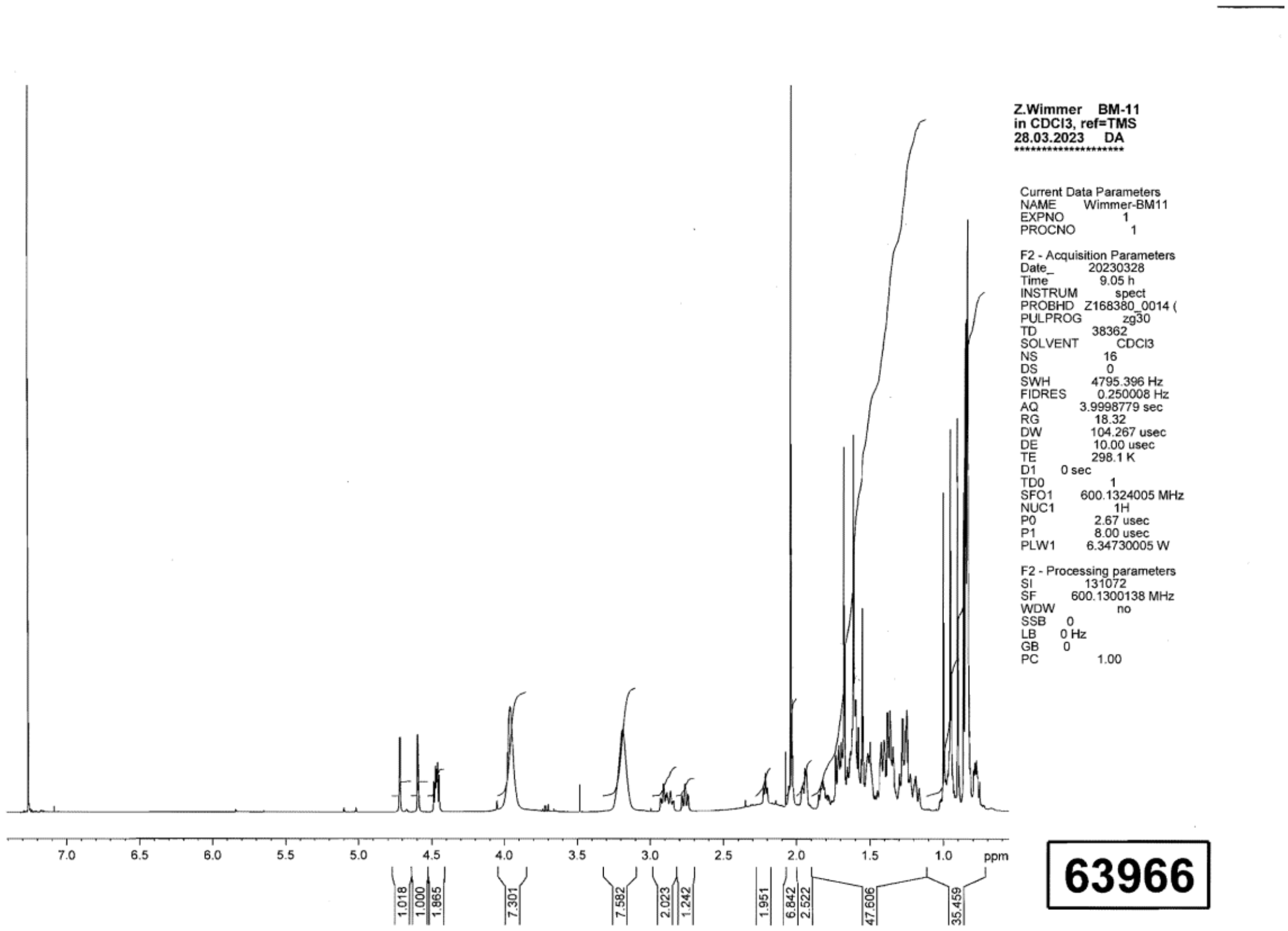


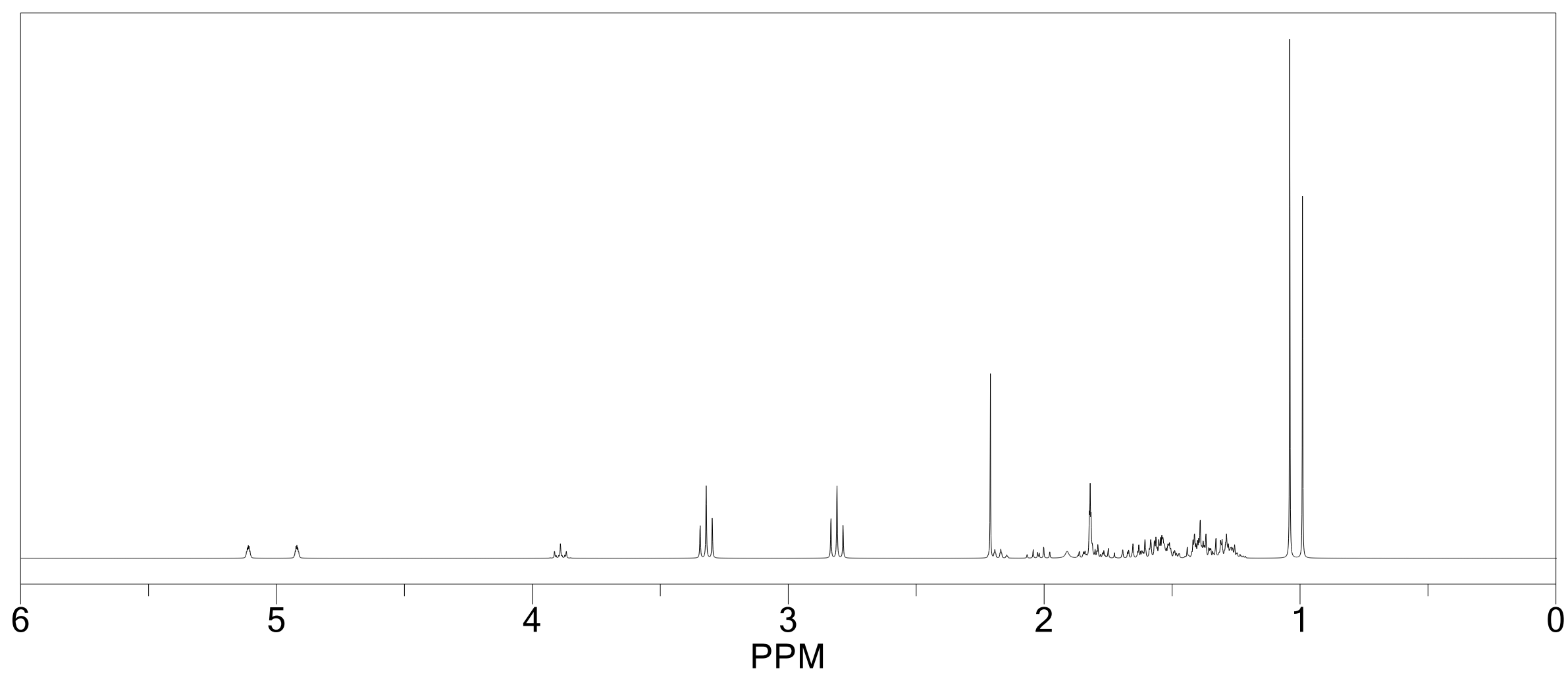

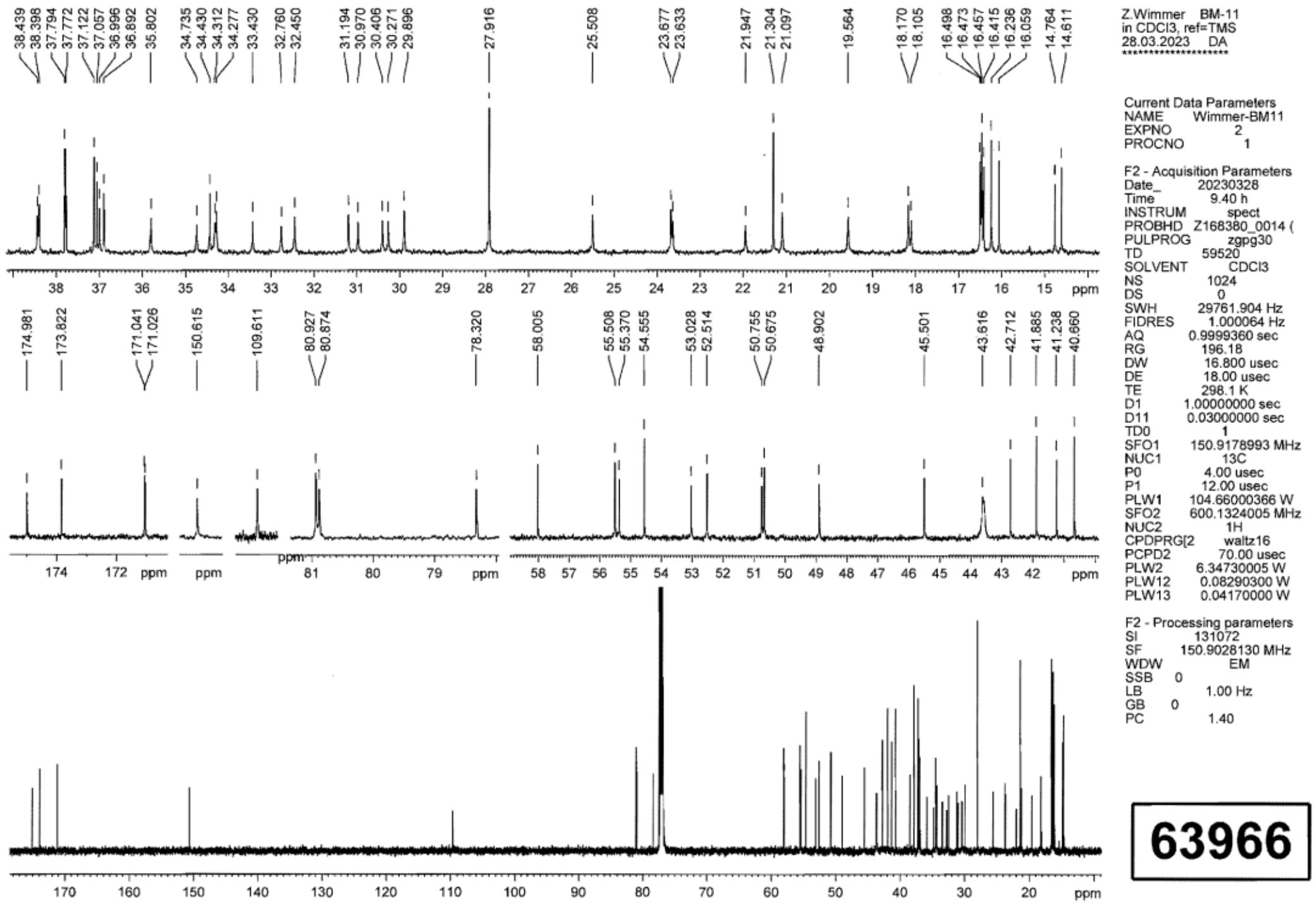
38.439 38.398 37.794 37.772 37.122 37.057 36.996 36.892 35.802 34.735 34.430 34.312 34.277 33.430 32.760 32.450 31.194 30.970 30.406 30.271 29.896 27.916 25.508 23.677 23.633 21.947 21.304 21.097 19.564 18.170 18.105 16.498 16.473 16.457 16.415 16.236 16.059 14.764 14.611
174.981 173.822 171.041 171.026 150.615 109.611 80.927 80.874 78.320 58.005 55.508 55.370 54.555 53.028 52.514 50.755 50.675 48.902 45.501 43.616 42.712 41.885 41.238 40.660
Z.Wimmer BM-11
in CDCl3, ref=TMS
28.03.2023 DA
Current Data Parameters
NAME Wimmer-BM11
EXPNO 2
PROCNO 1
F2 - Acquisition Parameters
Date_ 20230328
Time 9.40 h
INSTRUM spect
PROBHD Z168380_0014 (
PULPROG zgpg30
TD 59520
SOLVENT CDCl3
NS 1024
DS 0
SWH 29761.904 Hz
FIDRES 1.000064 Hz
AQ 0.9999360 sec
RG 196.18
DW 16.800 usec
DE 18.00 usec
TE 298.1 K
D1 1.00000000 sec
D11 0.03000000 sec
TD0 1
SFO1 150.9178993 MHz
NUC1 13C
P0 4.00 usec
P1 12.00 usec
PLW1 104.66000366 W
SFO2 600.1324005 MHz
NUC2 1H
CPDPRG[2 waltz16
PCPD2 70.00 usec
PLW2 6.34730005 W
PLW12 0.08290300 W
PLW13 0.04170000 W
F2 - Processing parameters
SI 131072
SF 150.9028130 MHz
WDW EM
SSB 0
LB 1.00 Hz
GB 0
PC 1.40
63966
ppm

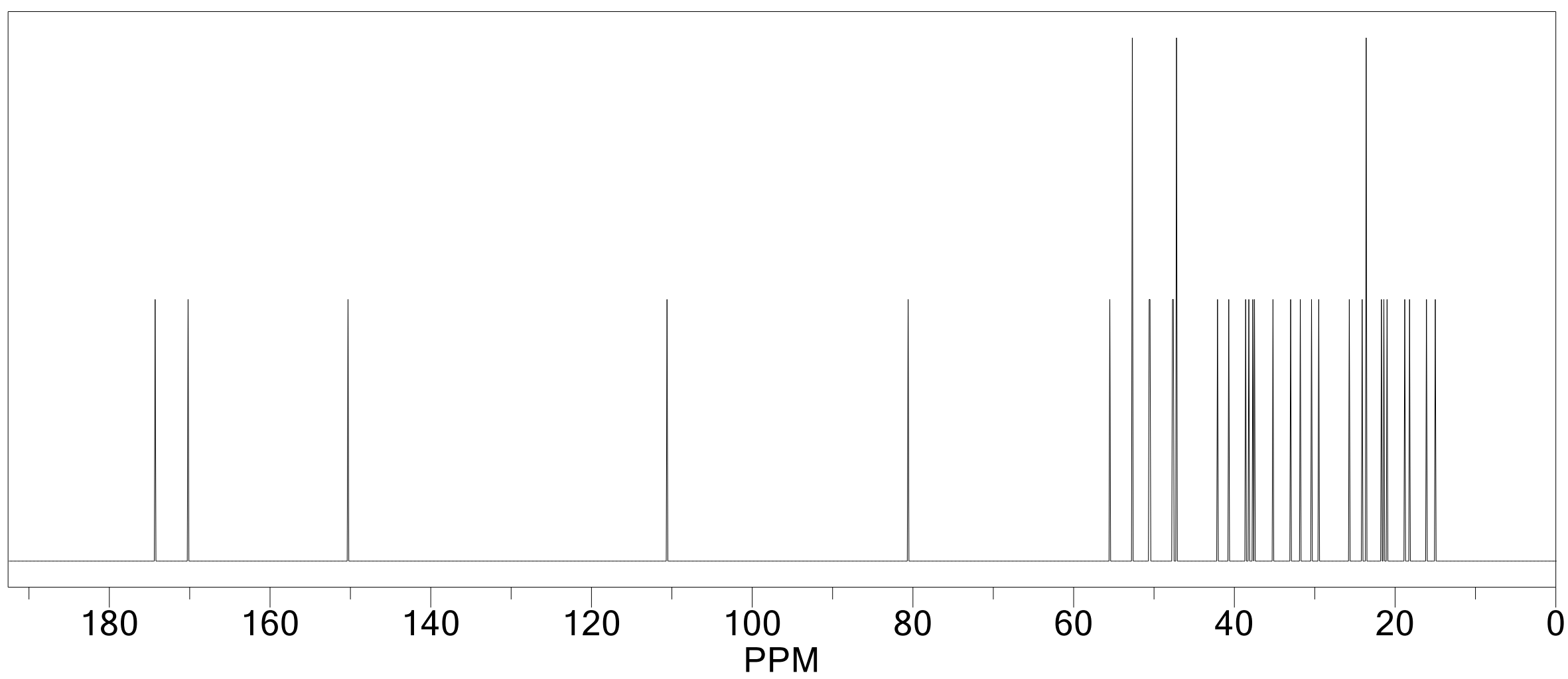
180 160 140 120 100 80 60 40 20 0
PPM

2.4. *tert*-Butyl (2-{4-[(3β)-3-acetyloxy-28-oxolup-20(29)-en-28-yl]piperazin-1-yl}-2-oxoethyl)carbamate (**5**) – **Figure S6**

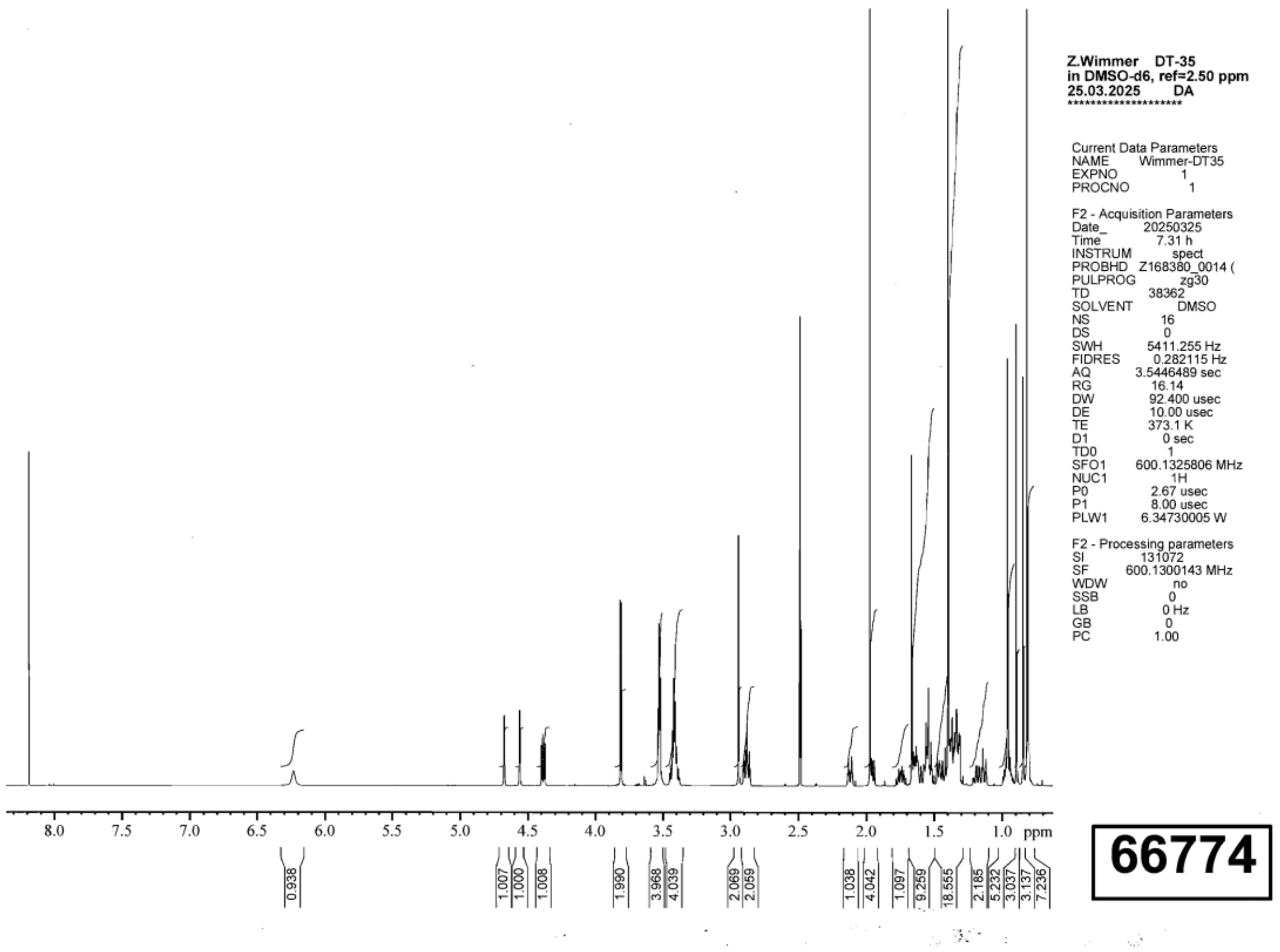


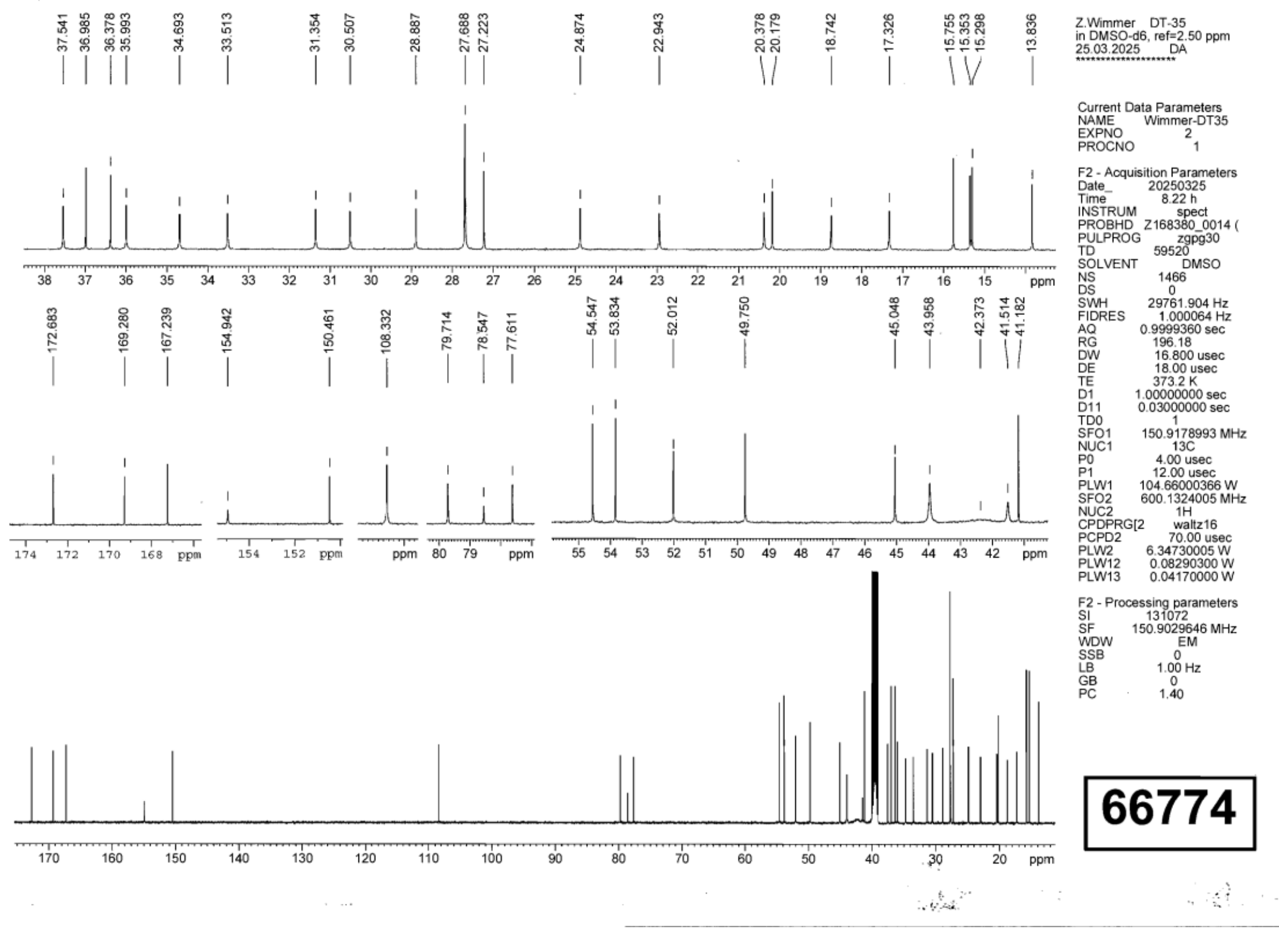

2.5. (3β)-28-(4-Glycylpiperazin-1-yl)-28-oxolup-20(29)-en-3-yl acetate (**6**) – **Figure S7**

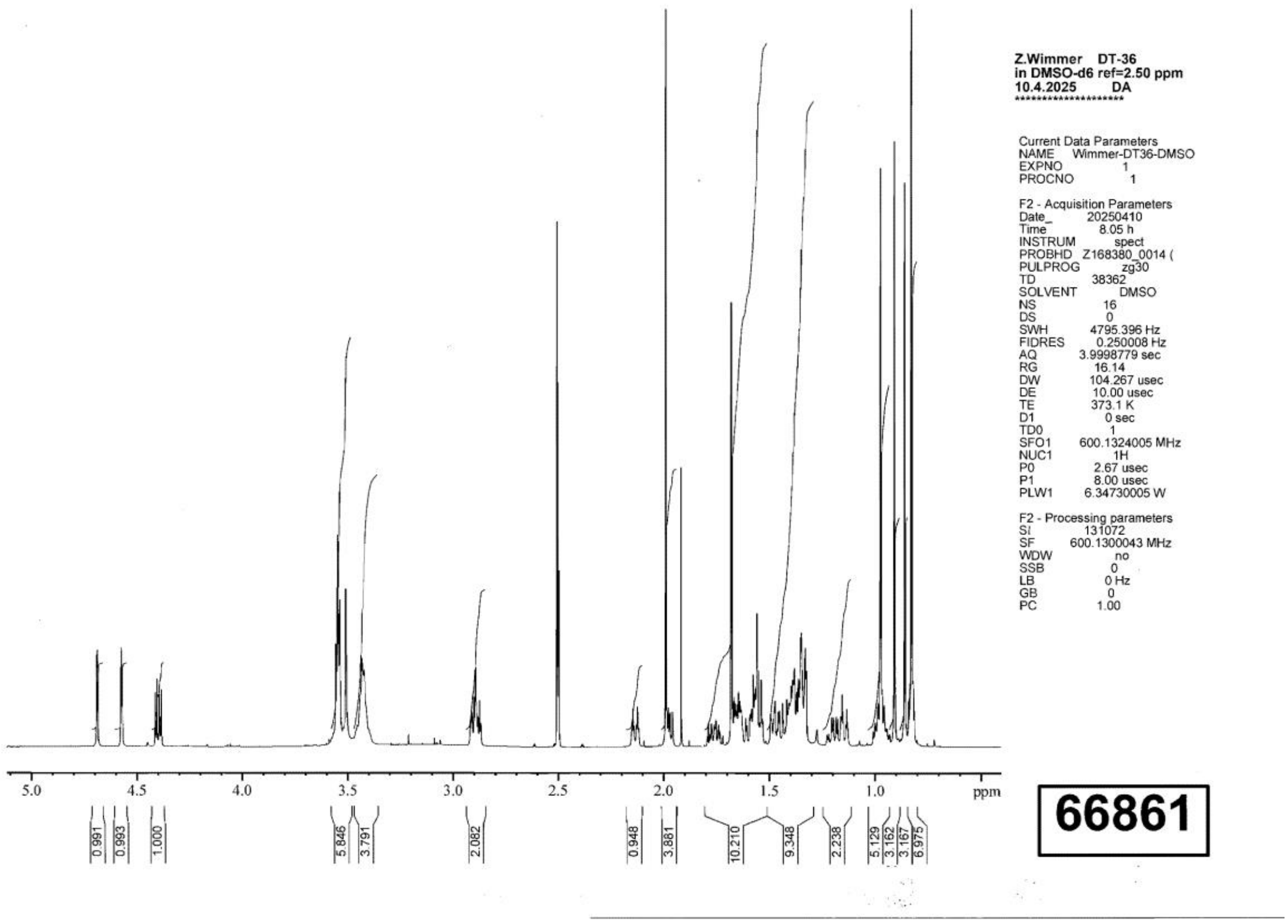


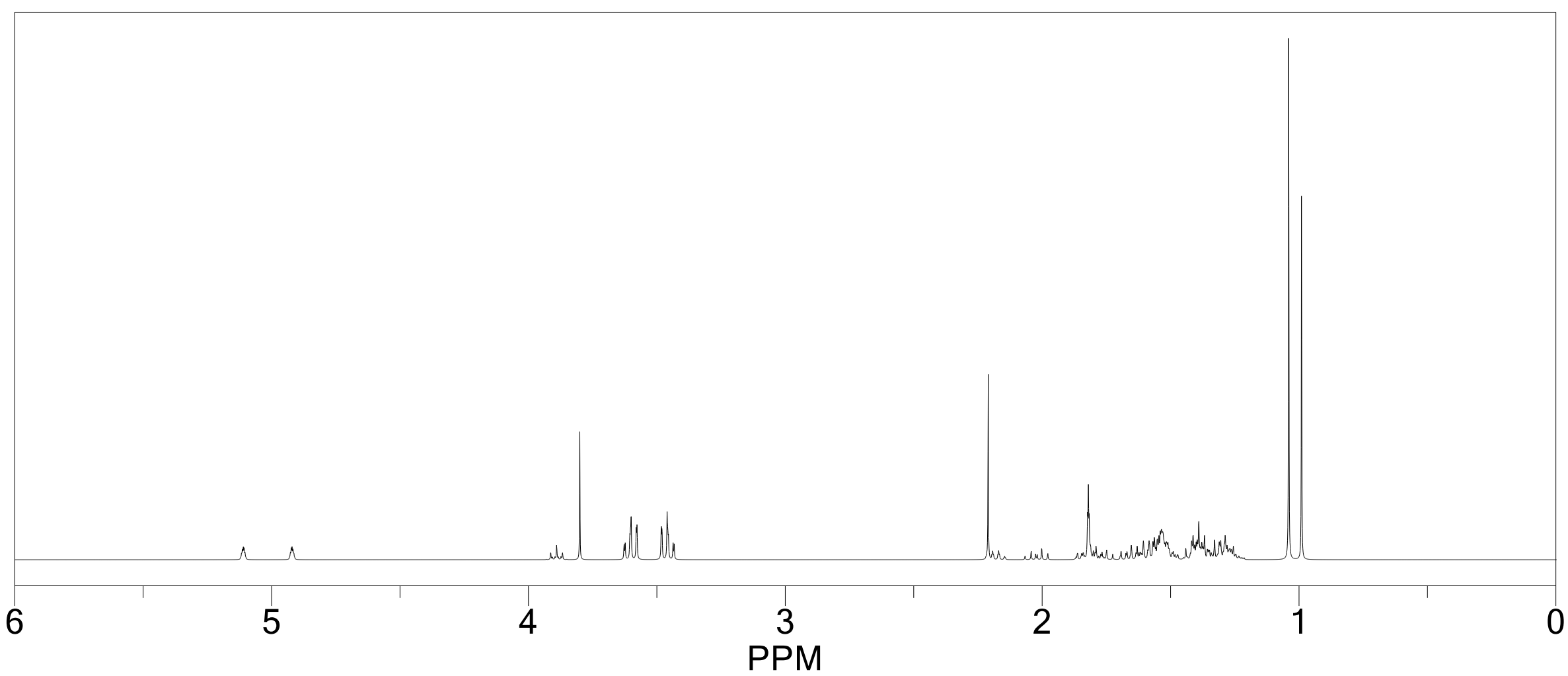

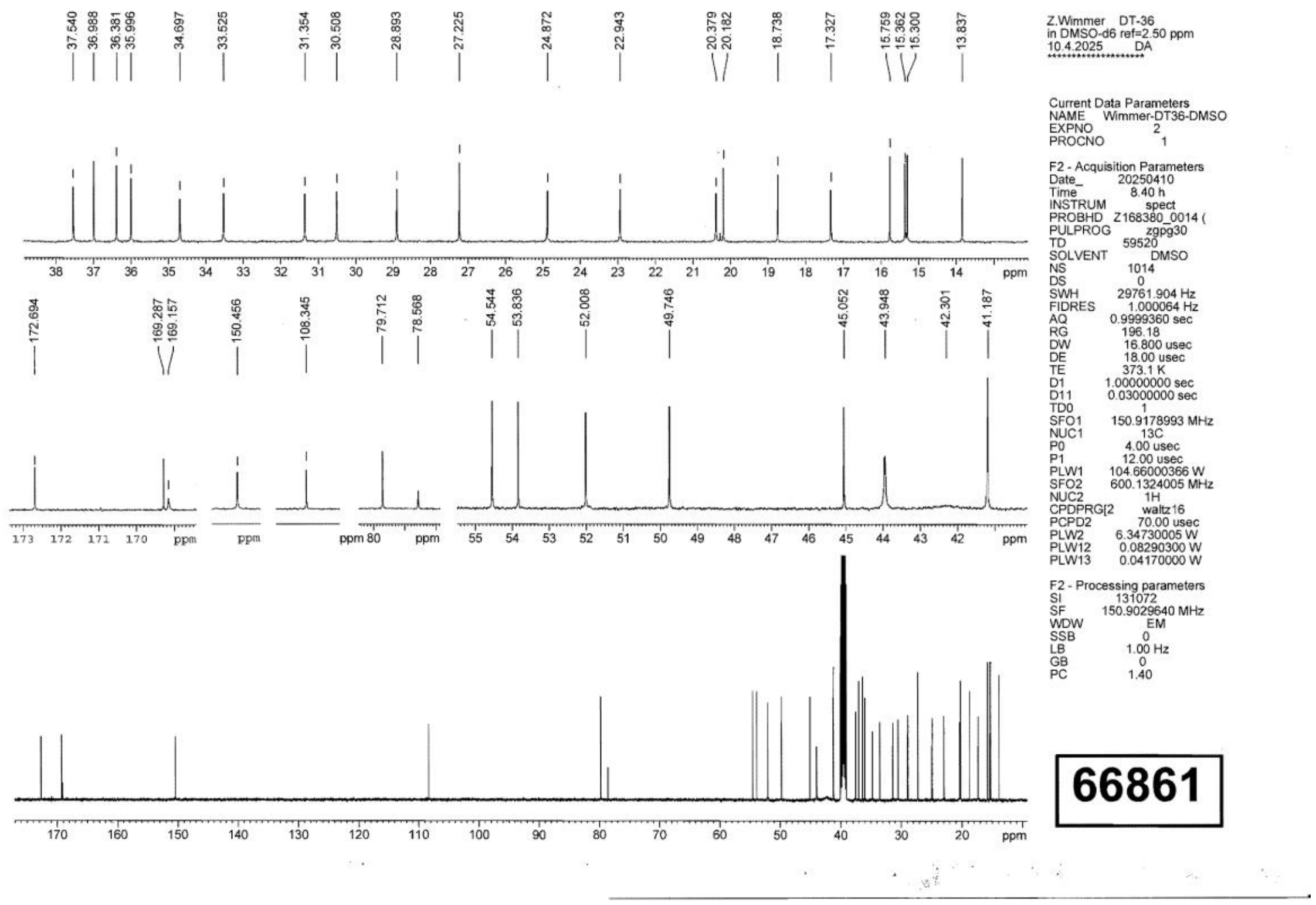
37.540
36.988
36.381
35.996
34.697
33.525
31.354
30.508
28.893
27.225
24.872
22.943
20.379
20.182
18.738
17.327
15.759
15.362
15.300
13.837
172.694
169.287
169.157
150.456
108.345
79.712
78.568
54.544
53.836
52.008
49.746
45.052
43.948
42.301
41.187
ppm
Z.Wimmer DT-36
in DMSO-d6 ref=2.50 ppm
10.4.2025 DA
Current Data Parameters
NAME Wimmer-DT36-DMSO
EXPNO 2
PROCNO 1
F2 - Acquisition Parameters
Date_ 20250410
Time 8.40 h
INSTRUM spect
PROBHD Z168380_0014 (
PULPROG zgpg30
TD 59520
SOLVENT DMSO
NS 1014
DS 0
SWH 29761.904 Hz
FIDRES 1.000064 Hz
AQ 0.9999360 sec
RG 196.18
DW 16.800 usec
DE 18.00 usec
TE 373.1 K
D1 1.00000000 sec
D11 0.03000000 sec
TD0 1
SFO1 150.9178993 MHz
NUC1 13C
P0 4.00 usec
P1 12.00 usec
PLW1 104.66000366 W
SFO2 600.1324005 MHz
NUC2 1H
CPDPRG[2 waltz16
PCPD2 70.00 usec
PLW2 6.34730005 W
PLW12 0.08290300 W
PLW13 0.04170000 W
F2 - Processing parameters
SI 131072
SF 150.9029640 MHz
WDW EM
SSB 0
LB 1.00 Hz
GB 0
PC 1.40
66861

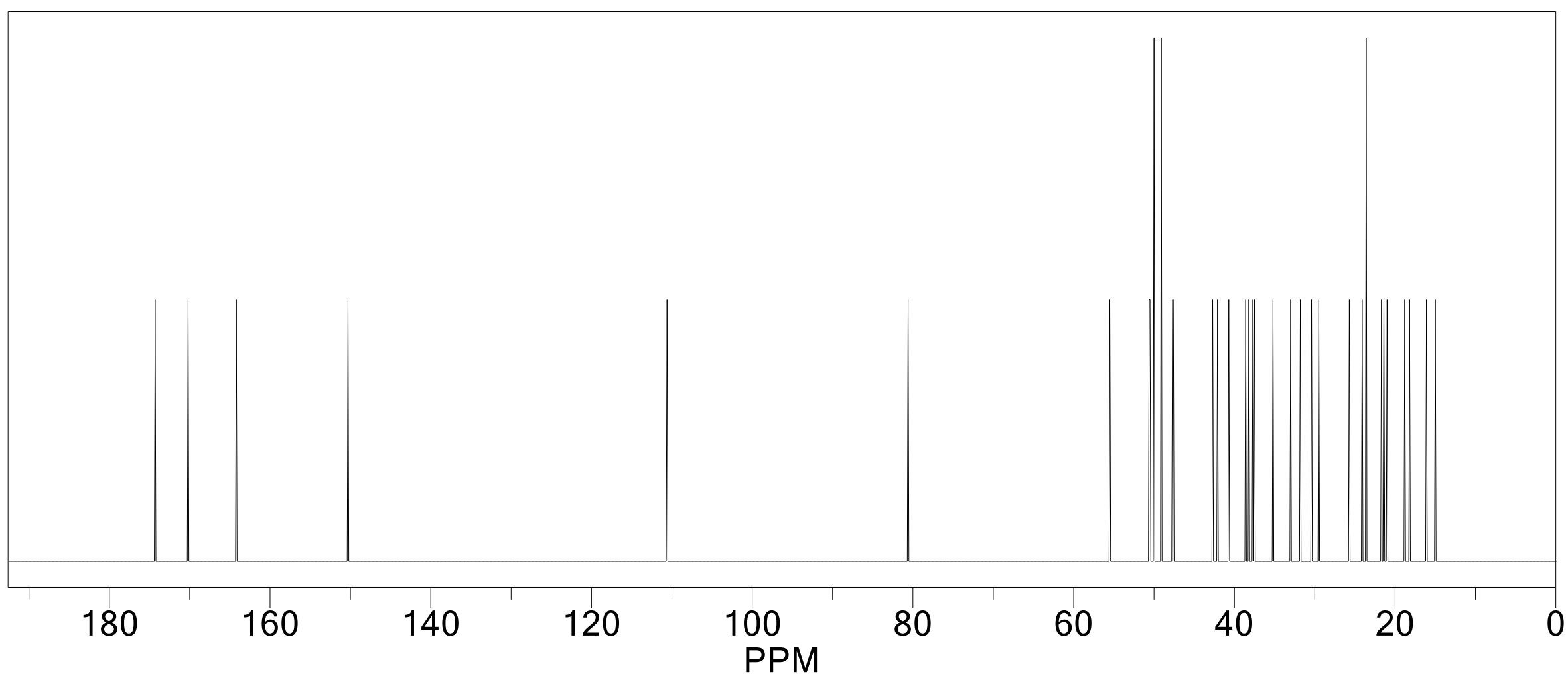
180
160
140
120
100
80
60
40
20
0
PPM

2.6. *tert*-Butyl (2-{4-[(3β)-3-hydroxy-28-oxolup-20(29)-en-28-yl]piperazin-1-yl}-2-oxoethyl)carbamate (**7**) – **Figure S8**

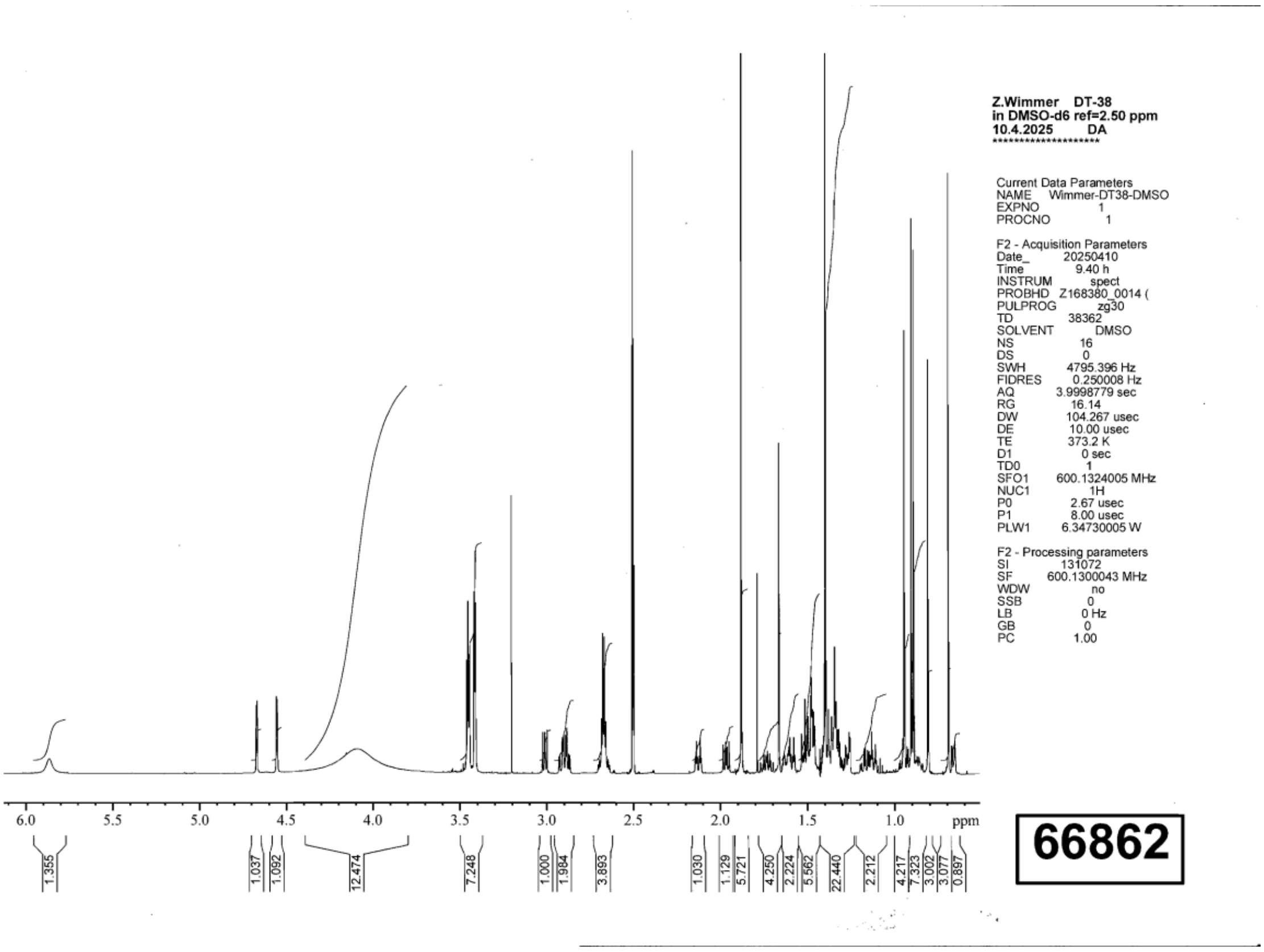


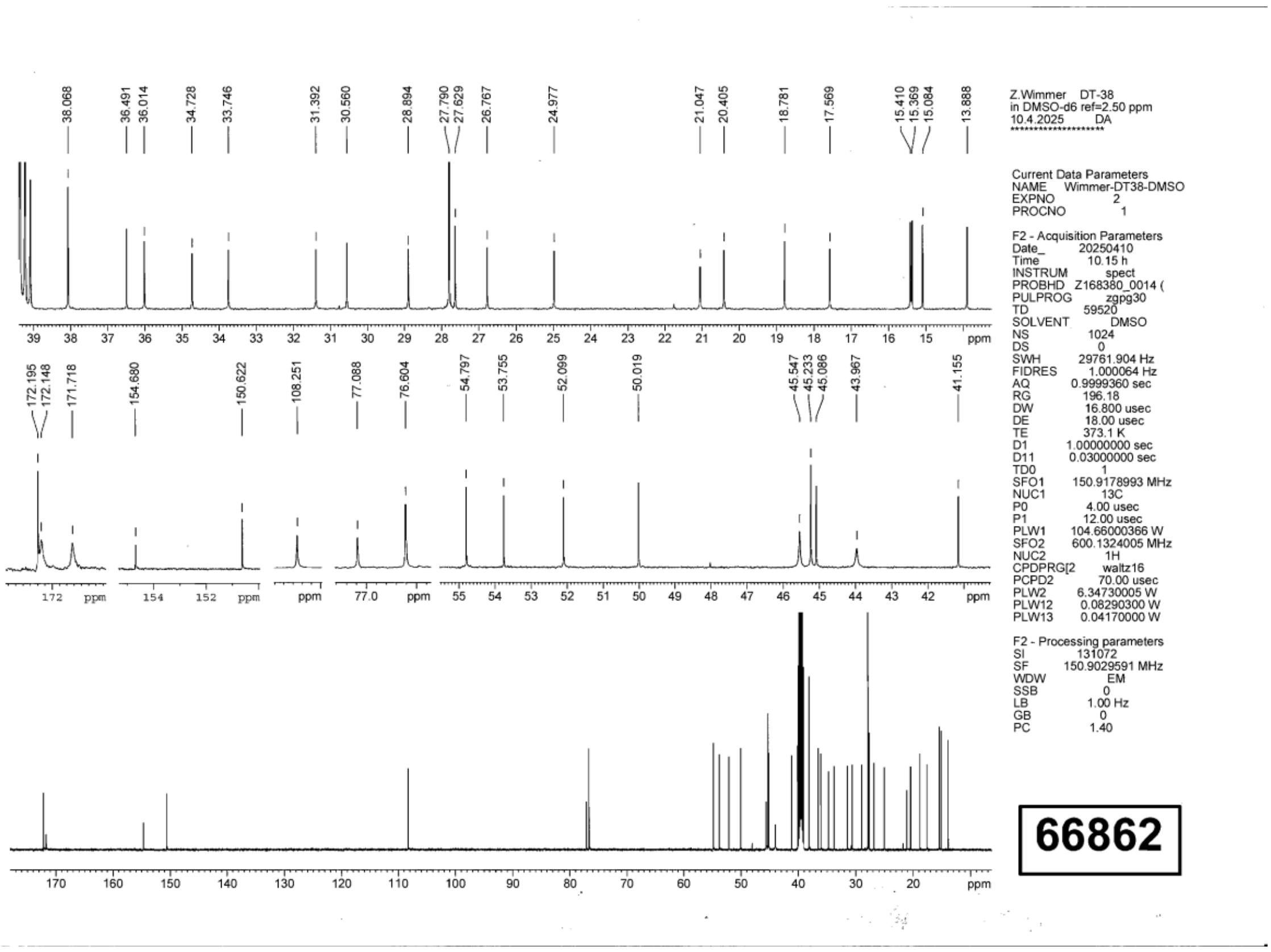

2.7. (3β)-28-[4-(Aminoacetyl)piperazin-1-yl]-3-hydroxylup-20(29)-en-28-one (**8**) – **Figure S9**

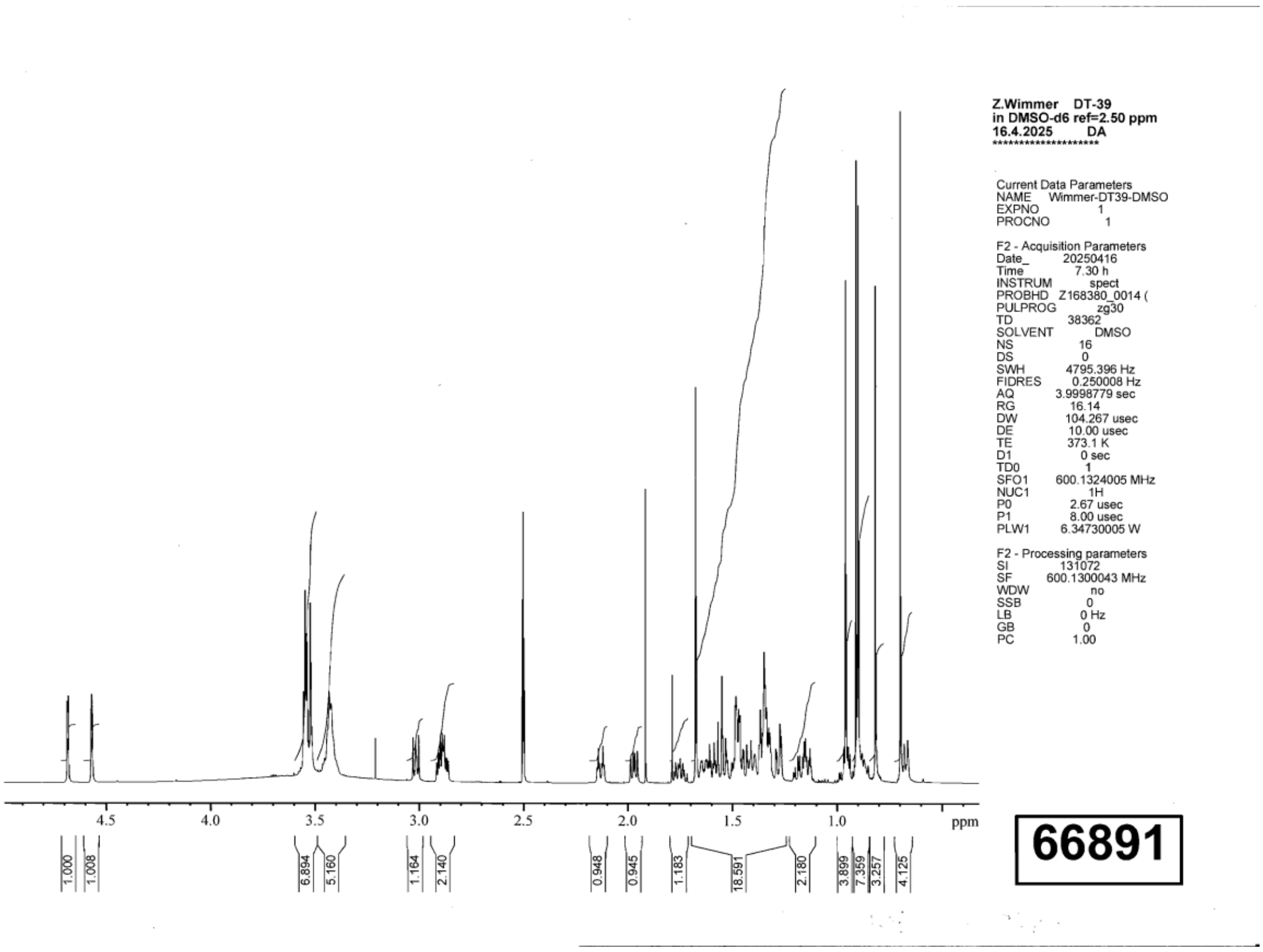


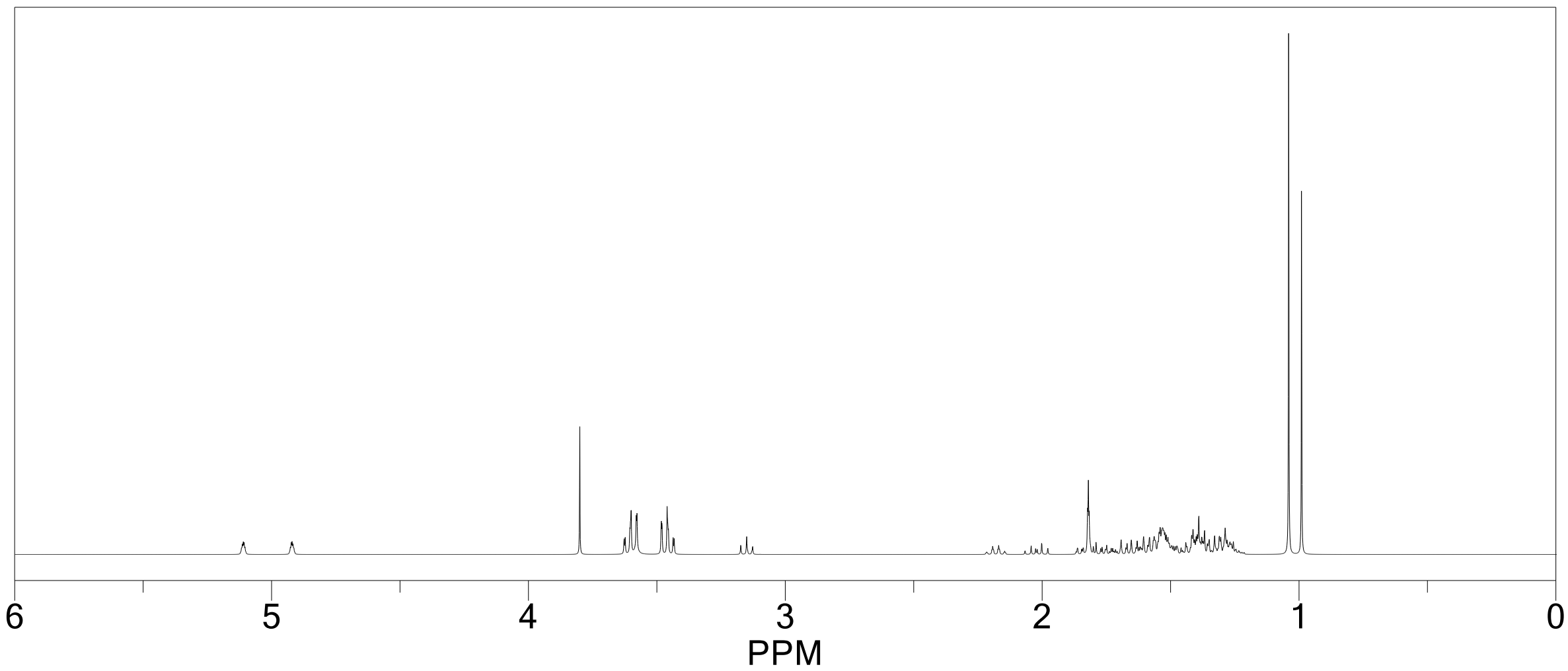

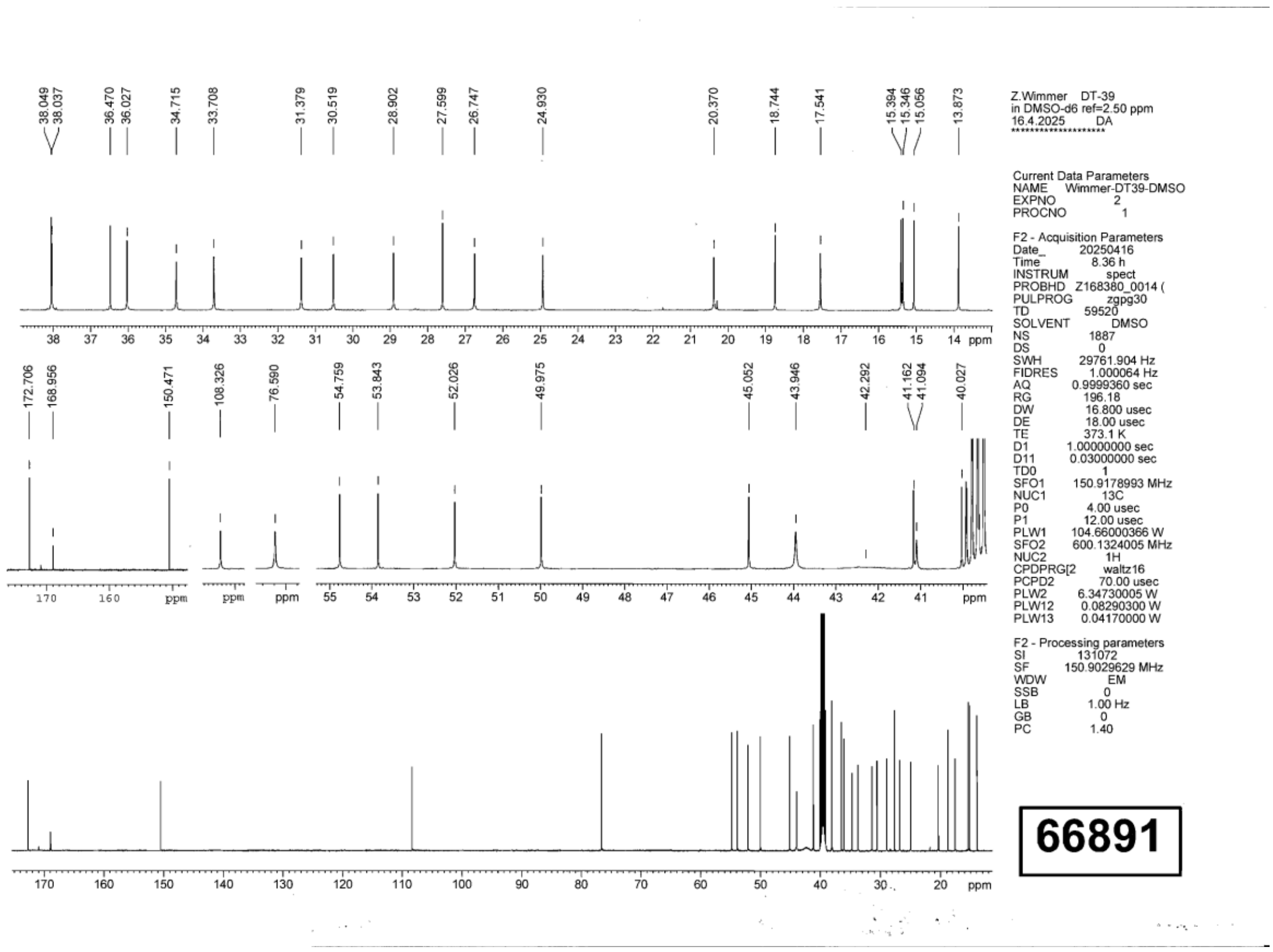
38.049
38.037
36.470
36.027
34.715
33.708
31.379
30.519
28.902
27.599
26.747
24.930
20.370
18.744
17.541
15.394
15.346
15.056
13.873
172.706
168.956
150.471
108.326
76.590
54.759
53.843
52.026
49.975
45.052
43.946
42.292
41.162
41.094
40.027
ppm
Z.Wimmer DT-39
in DMSO-d6 ref=2.50 ppm
16.4.2025 DA
Current Data Parameters
NAME Wimmer-DT39-DMSO
EXPNO 2
PROCNO 1
F2 - Acquisition Parameters
Date_ 20250416
Time 8.36 h
INSTRUM spect
PROBHD Z168380_0014 (
PULPROG zgpg30
TD 59520
SOLVENT DMSO
NS 1887
DS 0
SWH 29761.904 Hz
FIDRES 1.000064 Hz
AQ 0.9999360 sec
RG 196.18
DW 16.800 usec
DE 18.00 usec
TE 373.1 K
D1 1.00000000 sec
D11 0.03000000 sec
TD0 1
SFO1 150.9178993 MHz
NUC1 13C
P0 4.00 usec
P1 12.00 usec
PLW1 104.66000366 W
SFO2 600.1324005 MHz
NUC2 1H
CPDPRG[2 waltz16
PCPD2 70.00 usec
PLW2 6.34730005 W
PLW12 0.08290300 W
PLW13 0.04170000 W
F2 - Processing parameters
SI 131072
SF 150.9029629 MHz
WDW EM
SSB 0
LB 1.00 Hz
GB 0
PC 1.40
66891

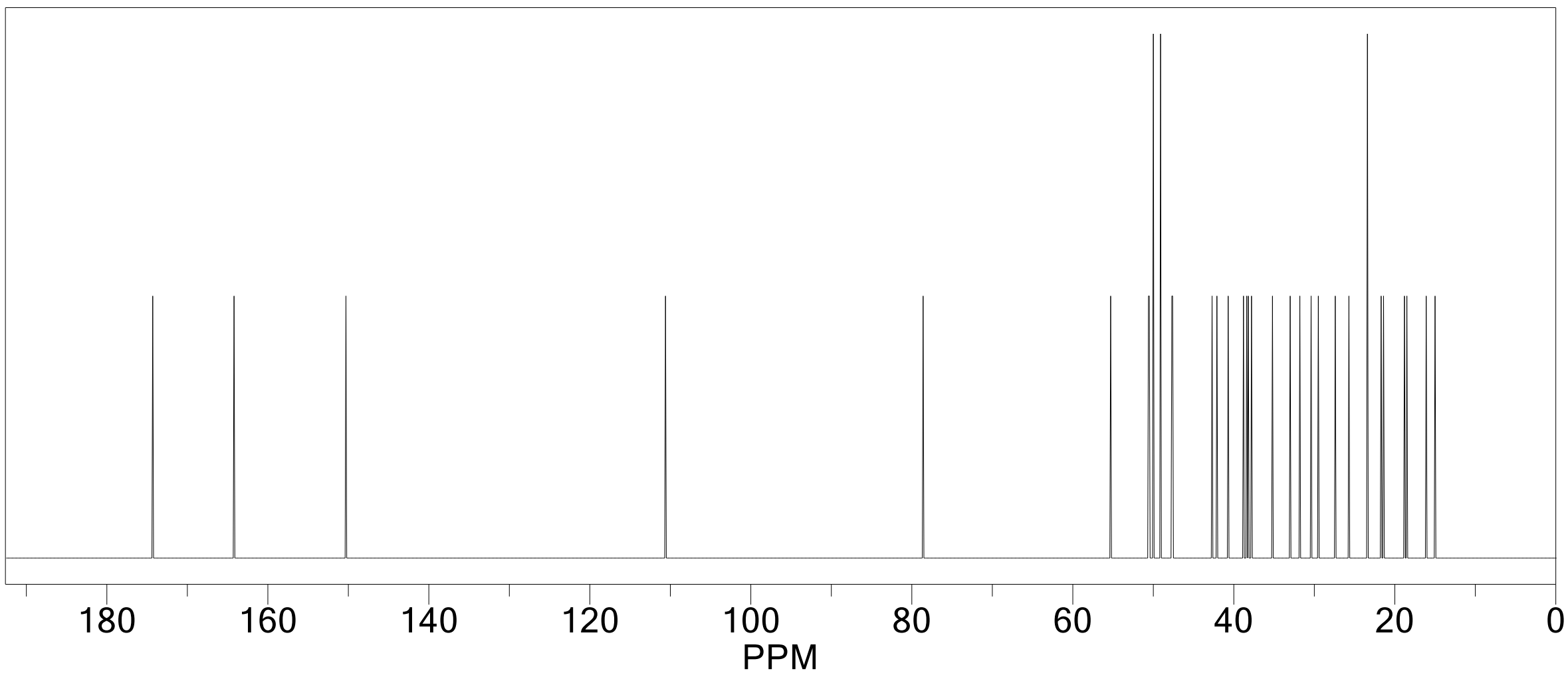
180
160
140
120
100
80
60
40
20
0
PPM
